# Reducing water use by alternate-furrow irrigation with livestock wastewater reduces antibiotic resistance gene abundance in the rhizosphere but not in the non-rhizosphere

**DOI:** 10.1016/j.scitotenv.2018.08.101

**Published:** 2019-01-15

**Authors:** Yuan Liu, Erping Cui, Andrew L. Neal, Xiaoxian Zhang, Zhongyang Li, Yatao Xiao, Zhenjie Du, Feng Gao, Xiangyang Fan, Chao Hu

**Affiliations:** a Farmland Irrigation Research Institute, Chinese Academy of Agricultural Sciences, Xinxiang 453002, China; b Department of Sustainable Agriculture Sciences, Rothamsted Research, Harpenden, Hertfordshire AL5 2JQ, UK

**Keywords:** Livestock wastewater, Alternate-furrow irrigation, Irrigation amount, Antibiotics resistance, Water quality

## Abstract

Livestock wastewater is rich in nutrients but may contain antibiotics and antibiotic resistance genes (ARGs). Their discharge to watercourses or soil may result in proliferation of ARGs. Irrigation with wastewater appears to be the most feasible option of disposing of it. One efficient irrigation technology used in arid regions is alternate-furrow irrigation (AFI) by alternately drying part of the plant roots for a prolonged period to physiologically reduce transpiration without compromising yield. However, the extent to which AFI with wastewater influences the concentration of antibiotics and spread of ARGs in soil is poorly understood. The purpose of this paper is to investigate how AFI using swine wastewater alters antibiotic kinetics and ARGs abundance under different irrigation rates, using pepper as the model plant. We examined three AFI treatments using 50%, 65% and 80% of the amount of water employed in sufficient conventional furrow irrigation. Each treatment had a groundwater irrigation control. The results showed that antibiotic concentrations and relative ARGs abundance in the top 20 cm of soil did not increase with the irrigation amount, although they were higher than those in the groundwater-irrigated soils. The relative ARGs abundance in the soil was modulated by irrigation amount and reducing the irrigation amount in AFI reduced ARGs dispersion only in rhizosphere. When the soil moisture was close to field capacity, ARGs were more abundant in rhizosphere than in non-rhizosphere, possibly because the rhizosphere is rich in microbes and increasing antibiotic concentrations due to an increase in irrigation rate favors antibiotic-resistant microbiome in competing for substrates. These, however, were not mirrored in the relative ARGs abundance in the roots. These results have important implications as it revealed that reducing the input of antibiotics and ARGs into soil with AFI does not necessarily reduce ARGs proliferation.

## Highlights

• First study of alternate-furrow irrigation effects on ARGs in soil
• Rhizosphere was more sensitive to water source than non-rhizosphere soil.
• Cd had greater influence on ARGs distribution than antibiotics.
• Sulfonomides had a greater influence on ARGs distribution than tetracyclines.
• Reducing irrigation amount with AFI reduced ARGs dispersion only in rhizosphere

## Graphical abstract

**Figure:**
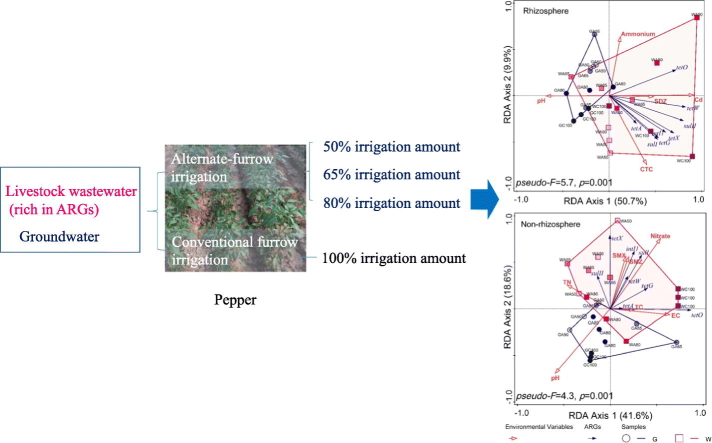

## 1 Introduction

Water used in agricultural production accounts for 50–80% of freshwater consumed globally (Palese et al., 2009). The combined pressures from agricultural production, increasing demand for water from population growth and global climate change have necessitated the use of recycled wastewater for agricultural irrigation to relieve water scarcity (Stroosnijder et al., 2012). Concurrently, livestock production is shifting towards large and more specialized farms, producing greater and more centralized quantities of wastes. For example, in China, >3 × 10^9^ tons of manures are produced each year (Xie et al., 2018). In Jiangsu Province of China alone, the overall output of livestock was 4.2 × 10^6^ tons in 2013, and the output of pork was 2.3 × 10^6^ tons and 2.3 × 10^6^ tons in 2013 and 2014, respectively (Wang et al., 2016). In United States, the total production of recoverable beef, dairy and swine manures was 37.7 × 10^6^ tons (dry weight) in 2016 (Milbrandt et al., 2018). There are potential benefits of using wastewater from livestock production for irrigation due to its richness of nutrients and it is also an effective way to reduce pollution resulting from arbitrary discharge of such wastes to the environment.

However, livestock wastewaters are reservoirs of both antibiotics and microbial antibiotic resistance genes (ARGs) (Qiao et al., 2018). For example, wastes from swine and chicken farms are shown to be associated with high concentrations of antibiotics and abundant antibiotic resistance genes (Lu et al., 2010; Sayah et al., 2005; Wei et al., 2011). Over the past few decades, development of large-scale, concentrated animal feeding operations has increased the extensive use of veterinary antibiotics for infection treatment, disease prevention and growth promotion. Daily global consumption of antibiotics had increased from 2000 to 2015 (Klein et al., 2018). In China alone, it is estimated that 53,800 tons of antibiotics entered the environment in 2013 even after waste treatment (Zhang et al., 2015). Residual antibiotics could exert selection pressure on environmental microorganisms, contributing to the spread of ARGs and antibiotic resistant microorganisms (Pruden et al., 2006). This pressure-driven spread of antibiotic resistance compromises the efficacy of antibiotics in animal and human medicine and is a global public health threat. The United Nations recently warned that antibiotic resistance is a crisis that cannot be ignored and has called for responsible use of antibiotics at the World Antibiotic Awareness Week held in 2017.

During irrigation with livestock wastewater, ARGs spread through soil, plants and surface runoff (Ghosh and LaPara, 2007; Joy et al., 2013). For example, compared to a wastewater-irrigated soil in summer, the total ARGs in soil fell 1.66 log-fold in winter when no irrigation was applied (Sui et al., 2016). Bastida et al. (2017) reported that both water quality and irrigation amount affected soil microbial communities in a semi-arid citrus orchard, and Mavrodi et al. (2018) found that irrigation influenced the overall diversity of a wheat rhizosphere microbiome and the relative abundance of specific operational taxonomic units (OTUs) in a three-year field irrigation experiment by altering soil water potential and pH. Ma et al. (2018) revealed that irrigation water sources affected accumulation and transport of pharmaceutical and personal care products (PPCPs) in vadose zone soils, but they did not consider ARGs.

Antibiotic resistance in soil spreads preferentially along water flow paths (Lüneberg et al., 2018) and ARGs dissemination depends on the mobility of individual antibiotic in soil. Santiago et al. (2016) found that high soil moisture resulted in high concentrations of PPCPs, including ofloxacin - a quinolone antibiotic - in irrigation with recycled wastewater, suggesting that the mobility of PPCPs in soil increased with soil moisture. Increasing irrigation frequency with reclaimed water was found to increase the level of ARGs in soil (Fahrenfeld et al., 2013). In addition to irrigation timing and amount, irrigation methods may also affect the spread of ARGs due to their potential influence on soil microorganisms, antibiotic distribution and other factors such as soil moisture, pH, organic matter and nutrients. However, to what extent these factors combine to modulate antibiotics and ARGs in soil-plant system is poorly understood.

Many irrigation methods have been developed to increase water use efficiency (WUE) in arid or semi-arid regions. Conventional furrow irrigation (CFI) is arguably the most traditional method despite its low WUE. Alternate-furrow irrigation (AFI) is a more efficient and easily implemented method by alternately irrigating two adjacent furrows to promote abscisic acid (ABA) synthesis by roots in the dry side in attempts to reduce stomatal conductance and thus transpiration (Graterol et al., 1993; Kang et al., 2000a; Kang et al., 2000b). AFI has been replacing CFI in most semiarid regions as the dominant irrigation method. The purpose of this study is to investigate the effects of AFI on the spread of antibiotics and ARGs in a pepper-cultivation field experiment using swine wastewater, with groundwater irrigation as control. We hypothesized that irrigation method, water quality and irrigation rates all influence the abundance of ARGs in soil. Our objective was to identify the associations between environmental factors and ARGs distribution in soil and plant tissues under AFI with livestock wastewater to improve our understanding of the environmental risks of irrigation with livestock wastewater and provide some reference information for safe use of livestock wastes in agricultural production.

## 2 Materials and methods

### 2.1 Soil

The experiment was carried out in a vinyl tunnel at the Agriculture Water and Soil Environmental Field Science Research Station, Chinese Academy of Agricultural Science at Xinxiang (Henan Province, 35°15′44″N, 113°55′6″E). The vinyl tunnel acted only to intercept rainwater and had no temperature, light, CO_2_ or moisture control. The field soil was a sandy loam (fluvo-aquic soil according to Chinese classification, Fluvic Cambisol according to the World Reference Base). The chemical properties of the top soil (0–20 cm) were: pH 8.5, electrical conductivity (EC) 87.7 mS m^−1^, organic matter (OM) 9.0 g kg^−1^, total N 0.7 g kg^−1^, nitrate‑nitrogen (NO_3_^−^-N) 136 mg kg^−1^, ammonium‑nitrogen (NH_4_^+^-N) 7.9 mg kg^−1^, available potassium (K) 252 mg kg^−1^, available phosphorus (P) 33.2 mg kg^−1^, total copper (Cu) 25.7 mg kg^−1^, total zinc (Zn) 72.4 mg kg^−1^, total lead (Pb) 22.0 mg kg^−1^, total cadmium (Cd) 0.60 mg kg^−1^, available Cu 1.5 mg kg^−1^, available Zn 1.8 mg kg^−1^, available Pb 1.9 mg kg^−1^, available Cd 0.20 mg kg^−1^.

### 2.2 Water

Groundwater and swine wastewater were used in our study. The groundwater was pumped to the field through plastic pipes with a flow meter from a depth of 4.5 m below the ground level at the experimental site. Swine wastewater was taken from a fermentation tank in a pig farm near the research station. The farm has an annual stock of about 3000 pigs, producing approximately 40,000 tons of wastewater annually. Water properties are presented in Table 1.

**Table 1:** Properties of groundwater and wastewater.

	pH	EC	COD^a^	TDS^b^	N	P	Ca	Mg	Fe	Zn	Mn
	–	μS cm^−1^	mg L^−1^	mg L^−1^	mg L^−1^	mg L^−1^	mg L^−1^	mg L^−1^	mg L^−1^	mg L^−1^	μg L^−1^
Groundwater	8.07	1985	104	2251	0.550	–	55.5	122	1.07	0.021	178
Wastewater	8.38–8.42	2570–2605	311–349	1707–1655	262–389	15.8–17.4	46.3–48.9	32.4–44.7	0.85–0.92	0.26–0.47	118–121


	Pb	Cd	Cu	Cr	As	Hg	NO_3_^−^	PO_4_^3−^	SO_4_^2−^	K^+^	Na^+^
	μg L^−1^	μg L^−1^	μg L^−1^	μg L^−1^	μg L^−1^	μg L^−1^	mg L^−1^	mg L^−1^	mg L^−1^	mg L^−1^	mg L^−1^
Groundwater	0.654	0.050	2.45	13.3	9.85	0.065	–	–	844	2.95	514
Wastewater	1.63–1.83	0.086–0.127	63.3–83.0	25.5–34.5	1.90–2.31	0.170–0.186	2.31–3.09	3.73–6.16	295–343	199–226	235–279

Note: The content of N, P, Ca, Mg, Fe, Zn, Mn, Cu, Pb, Cd, Cr, As, Hg refers to the total content.

a Chemical oxygen demand.

b Total dissolved solids.

### 2.3 Plant cultivation

Pepper (*Capsicum annuum* L., Fulong F1) was cultivated as the model crop, as it is a vegetable eaten regularly and is often cultivated in vinyl tunnels. A mixture of perlite and vermiculite (1:1 w/w) was used as the seedling medium, which was then transferred into a seedling-nursing disk (4 × 8 cavaties, 5.3 cm in top diameter, 2.7 cm in bottom diameter, 5.8 cm in height, and a small hole at the bottom). Pepper seeds were sown into the prepared medium on April 14, 2017 and provided with Hoagland and Amon nutrient solutions (708 mg L^−1^ Ca(NO_3_)_2_·4 H_2_O, 1011 mg L^−1^ KNO_3_, 230 mg L^−1^ NH_4_H_2_PO_4_, 493 mg L^−1^ MgSO_4_·7H_2_O, 40 mg L^−1^ NaFe-EDTA, 2.86 mg L^−1^ H_3_BO_3_, 2.13 mg L^−1^ MnSO_4_·4 H_2_O, 0.22 mg L^−1^ ZnSO_4_·7 H_2_O, 0.08 mg L^−1^ CuSO_4_·5 H_2_O, 0.02 mg L^−1^ (NH_4_)_6_Mo_7_O_24_·4 H_2_O). After one month, healthy and uniform-sized seedlings were selected and transplanted to the field plots. Rows were spaced 50 cm apart and plants were spaced 50 cm apart along each row. There were 3 ridges of pepper plants and 4 furrows in each plot. Furrow depth was 30 cm. To ensure survival of the transplanted seedlings, each plot was watered with 400 L of groundwater (250 m^3^ ha^−1^) via CFI immediately after the transplantation, based upon the local farmers' established practice.

### 2.4 Field experiment

Before transplantation, the soil was applied with a base fertilizer consisting of 180 kg CO(NH_2_)_2_ ha^−1^, 450 kg Ca(H_2_PO_4_)_2_·H_2_O ha^−1^, and 240 kg KCl ha^−1^. A top dressing of 90 kg CO(NH_2_)_2_ ha^−1^ was applied on July 21, August 12 and September 3 respectively: the total amount of applied CO(NH_2_)_2_ was 450 kg ha^−1^. Each plot was 2 × 8 m in size, and there were 50-cm intervals between every two plots to avoid the interaction of water between adjacent plots. 400 L of groundwater via CFI was irrigated to each plot every 7 days until June 19, and groundwater or swine wastewater at different irrigation amounts were then employed until August 23. To reduce salt stress to the plants, wastewater was mixed with equal volume of groundwater prior to irrigation. The treatments were as follows:

1. GC100 (CFI with 100% of 400 L groundwater about every 10 days)
2. GA50 (AFI with groundwater, using 50% of GC100)
3. GA65 (AFI with groundwater, using 65% of GC100)
4. GA80 (AFI with groundwater, using 80% of GC100)
5. WC100 (CFI with wastewater of GC100 about every 10 days)
6. WA50 (AFI with wastewater, using 50% of WC100)
7. WA65 (AFI with wastewater, using 65% of WC100)
8. WA80 (AFI with wastewater, using 80% of WC100)

Each treatment had three replicates and all treatments were irrigated approximately at the same time on the same day. Comparison of CFI at 100% rate and AFI at 50% rate was used to examine the effects of AFI according to Kang et al. (2000b), and the 50%, 65% and 80% rates were compared to establish the optimal AFI rate which is effective in reducing ARGs dispersion. A bare plot with base fertilizer application but without cultivation and irrigation (BK) was also included to elucidate any changes in ARGs abundance in the soil not associated with cropping and irrigation.

To ensure a good yield, all plots were irrigated with 400 L of groundwater using CFI every 7 days from August 23 to harvest on October 9. The harvested plants were separated into root, stem, leaf and fruit tissues. At the same time, the topsoil (0–20 cm) of each treatment was sampled; soil shaken off the roots at harvest was considered as non-rhizosphere (NRS) while soil adhering to the roots was brushed off and collected as rhizosphere (RS). Soil collected from 5 randomly selected plants was pooled for each plot. For BK plots, three replicate soil samples were collected. Sampled plants were washed thoroughly with sterile saline solution (8.5 g L^−1^ NaCl) to remove adhering soil particles and surface microbes. Sub-samples of the soil and plant samples were stored at −80 °C, and the rest was air- or oven-dried before determination of their chemical properties (see description in the Supplementary Information).

### 2.5 Antibiotic compounds analysis

Six antibiotic compounds typically used in livestock production (Tang et al., 2015) were determined in our study according to the procedure of Cheng et al. (2016), with some minor modifications. These compounds were tetracycline (TC), chlortetracycline (CTC), oxytetracycline (OTC), sulfadiazine (SDZ), sulfamethoxazole (SMX) and sulfamerazine (SMZ). Details of the antibiotics determination are provided in the Supplementary Information.

### 2.6 DNA extraction

FastDNA SPIN Kits (MP Biomedicals, CA) were used to extract total DNA from soil, plant and water samples. Plant tissues were ground in liquid nitrogen before extraction. To determine the concentration and quality of the extracted DNA, spectrophotometric analysis (NanoDrop ND-2000c, Thermo Fisher Scientific, Waltham, MA) and 1.5% agarose gel electrophoresis were used. The bacterial 16S rRNA gene was amplified by PCR and sequenced by pyrosequencing. Detailed procedures are described in the Supplementary Information.

### 2.7 Relative quantification of ARGs and *intI1*

Seven ARGs (*tetA*, *tetG*, *tetO*, *tetW*, *tetX*, *sulI* and *sulII*), the class 1 integron integrase, *intI1*, and the 16S rRNA gene were amplified and quantified using quantitative polymerase chain reaction (qPCR) using a SYBR Green approach at Shanghai Personal Biotechnology Co., Ltd. (Shanghai, China). The procedure details are described in the Supplementary Information.

### 2.8 Statistical analysis

Statistical comparison of antibiotic resistance gene abundance and environmental parameters was performed using the software package SPSS 16.0 for Windows (SPSS Inc., Chicago, IL, USA). Mean differences between treatments were assessed by analysis of variance (ANOVA). *Post-hoc* pairwise comparisons of the treatment-means were performed using Duncan's multiple range test. Differences were considered significant at *p* < 0.05. Correlation tests were performed using Pearson's correlation coefficient.

MicrobiomeAnalyst (Dhariwal et al., 2017) was used to analyse the OTU data. A minimum mean abundance of 14 across all treatments was used as a cut-off, together with a low variance filter to remove those OTUs associated with the lowest 10% of the coefficients of variance, determined using the inter-quantile range. Abundance data was scaled using Cumulative Sum Scaling (CSS) (Weiss et al., 2017). Principal coordinate analysis (PCoA) of soil bacterial communities at the OTU-level was used, based on weighted UniFrac phylogenetic distance (Lozupone et al., 2011). We also employed hierarchical bi-clustering of OTUs associated with the different treatments and Ward's minimum variance method to identify clusters. To test for significant OTU assemblage divergence between different soils, we used permutation multivariate analysis of variance (PERMANOVA) based again on OTU weighted UniFrac distance. Where significant divergence between assemblages was detected, we also tested for homogeneity of multivariate dispersion between groups using PERMDISP (Anderson and Walsh, 2013). Where no significant difference in the multivariate dispersion was observed, we assumed the significant effects observed in PERMANOVA were ascribed to treatment.

Differences between relative antibiotic resistance and integron integrase gene abundances were assessed using Principal Coordinate Analysis (PCoA) based upon Gower distances (Kuczynski et al., 2010) in PAST 3.20. Two-factor PERMANOVA with 9999 Monte Carlo permutations was conducted in PAST to evaluate the divergence of ARGs between different treatments also using Gower distances. Where significant treatment effects were identified, Redundancy Analysis (RDA) was used to assess the relationships between ARGs abundance and environmental factors in CANOCO 5 (ter Braak, 1988). For each RDA model, interactive-forward-selection of environmental variables was used to identify the predictors of ARGs abundance. Before analysis, all environmental variables were transformed to *z*-scores. Statistical significance of each RDA model was assessed based upon 999 Monte Carlo permutations.

## 3 Results

### 3.1 Concentrations of antibiotics and relative abundance of ARGs and *intI1* in irrigation waters

We measured the concentration of six antibiotic compounds in the wastewater and groundwater used to irrigate the plots and the abundance of seven ARGs and *intI1* in wastewater relative to groundwater. The concentration of TC, CTC, OTC, SMX, SMZ and SDZ in the groundwater varied in the range of 7.1–2.1, 9.0–2.2, 15.7–8.2, 5.0–3.7, 6.1–4.1 and 3.1–2.2 ng L^−1^, respectively, and in the wastewater changed in the range of 354.2–126.5, 311.4–184.7, 5471.3–1136.5, 5.1–4.9, 4.6–4.1 and 9.2–3.0 ng L^−1^, respectively. Tetracycline concentrations in the wastewater were significantly higher than tetracyclines in the groundwater and sulfonamides in the wastewater. Compared to their abundances in the groundwater, *tetA*, *tetG*, *tetO*, *tetW*, *tetX*, *sulI*, *sulII* and *intI1* genes in the wastewater were 9.2–14.2, 409.0–176.1, 30.8–3.7, 634.3–149.7, 11.0–3.5, 183.8–88.0, 1357.0–361.8 and 28.9–6.8 fold more abundant, respectively.

### 3.2 The chemical properties of soil following irrigation

Soil chemical properties, including pH, EC, OM, total nitrogen, NO_3_^−^-N, NH_4_^+^-N and bioavailable heavy metals, measured after harvest are presented in Figs. S1 and S2. The pH and bioavailable Zn were higher in the groundwater-irrigated soils, while OM, total nitrogen, NO_3_^−^-N and bioavailable Cd were higher in the wastewater-irrigated soils. EC and NO_3_^−^-N were higher in the non-rhizosphere than in the rhizosphere, as opposed to OM and NH_4_^+^-N.

### 3.3 Concentrations of antibiotic compounds in soil

#### 3.3.1 Water quality and alternate-furrow irrigation rate effects

**Fig. 1:**
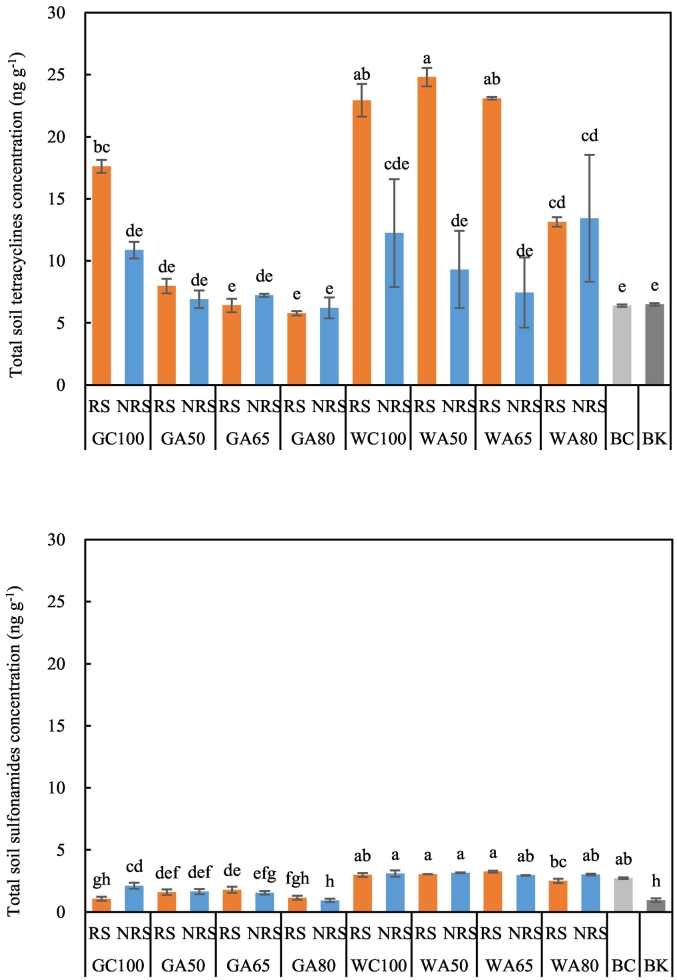
The concentration of antibiotic compounds in irrigated and unirrigated soils. The concentration of tetracyclines is the sum of the concentrations of tetracycline, chlortetracycline and oxytetracycline (Table S3). The concentration of sulfonamides is the sum of the concentrations of sulfadiazine, sulfamethoxazole and sulfamerazine (Table S3). RS refers to rhizosphere, NRS refers to non-rhizosphere, G refers to groundwater, W refers to livestock wastewater, C refers to conventional furrow irrigation, A refers to alternate-furrow irrigation. 100, 50, 65 and 80 refer to 100%, 50%, 65% and 80% of full irrigation amount per plot, respectively. BC refers to the original soil before fertilization and cultivation, BK refers to bare plot soil with base fertilizer only but no cultivation and no irrigation. The data are expressed as the mean ± standard deviation of concentration based on 3 replicates. Lower-case letters above each column denote groups between which significant differences occur at *p* < 0.05 determined from Duncan's post-hoc pairwise comparisons.

Sulfonamide concentrations in soil were much lower than that of tetracyclines (Fig. 1), following the pattern observed in the irrigation waters. Wastewater irrigation resulted in higher concentrations of antibiotics in soil than groundwater irrigation. There was no significant increase in antibiotics concentrations in either rhizosphere or non-rhizosphere with irrigation rate increasing under AFI.

#### 3.3.2 Alternate-furrow irrigation effects

Under wastewater irrigation, there were no significant differences in concentrations of the antibiotics between CFI and AFI at the 50% rate in either rhizosphere or non-rhizosphere. Comparison of AFI and CFI under groundwater irrigation indicated that AFI significantly reduced tetracycline concentrations while significantly increased sulfonamides in the rhizosphere, suggesting that AFI did not have a consistent effect upon different classes of antibiotics in soil.

#### 3.3.3 Other effects

There were no significant differences in sulfonamide concentrations between the original soil and the wastewater-irrigated soils, or between the rhizosphere and the non-rhizosphere. Irrigation with groundwater did not increase the concentration of antibiotics in soil significantly, except in the rhizosphere under CFI. For tetracyclines, wastewater irrigation resulted in a significant increase in their concentrations in the rhizosphere under all treatments compared to the original and unirrigated soil. Under CFI with either water source, the concentrations of tetracycline compounds were significantly higher in the rhizosphere than in the non-rhizosphere, while under AFI, this occurred only with wastewater at the 50 and 65% rates. When the irrigation rate under AFI increased to 80%, however, the difference in concentrations of the antibiotics between the rhizosphere and the non-rhizosphere disappeared. The concentrations of sulfonamides in the soil of the bare plot were lower than that in the original soil, while tetracyclines remained unchanged.

### 3.4 Bacterial community composition

**Fig. 2:**
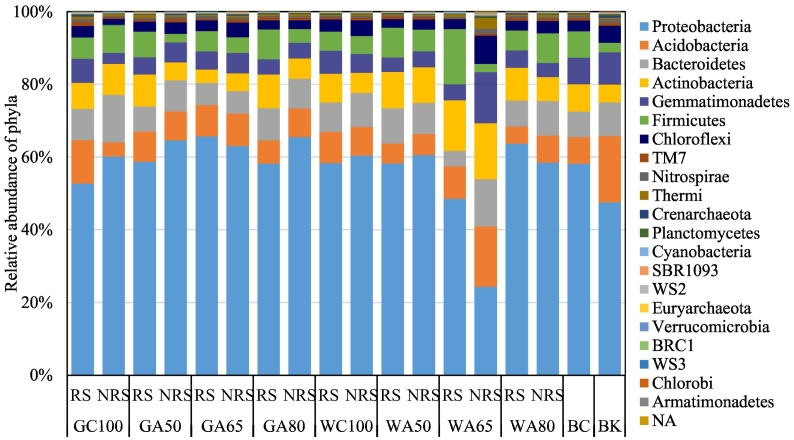
The relative abundance of soil bacterial phyla in irrigated and unirrigated soils. RS refers to rhizosphere, NRS refers to non-rhizosphere, G refers to groundwater, W refers to livestock wastewater, C refers to conventional furrow irrigation, A refers to alternate-furrow irrigation. 100, 50, 65 and 80 refer to 100%, 50%, 65% and 80% of full irrigation amount per plot, respectively. BC refers to the original soil before fertilization and cultivation, BK refers to bare plot soil with base fertilizer only but no cultivation and no irrigation.

Overall, 2626 OTUs were identified in a total of 3,488,396 amplicon sequences (average sequences per sample 72,674; range 94,028-40,558). Dominant phyla in the soils were Proteobacteria, Acidobacteria, Bacteroidetes, Actinobacteria, Gemmatimonadetes, Firmicutes and Chloroflexi, which together accounted for over 93% of all OTUs (Fig. 2). The relative abundance of Actinobacteria and Firmicutes was higher in wastewater-irrigated soils than groundwater-irrigated soils, but not significantly. Compared with that in the original soil, Actinobacteria, Gemmatimonadetes, Bacteroidetes and Chloroflexi increased in the soil of the bare plot.

**Fig. 3:**
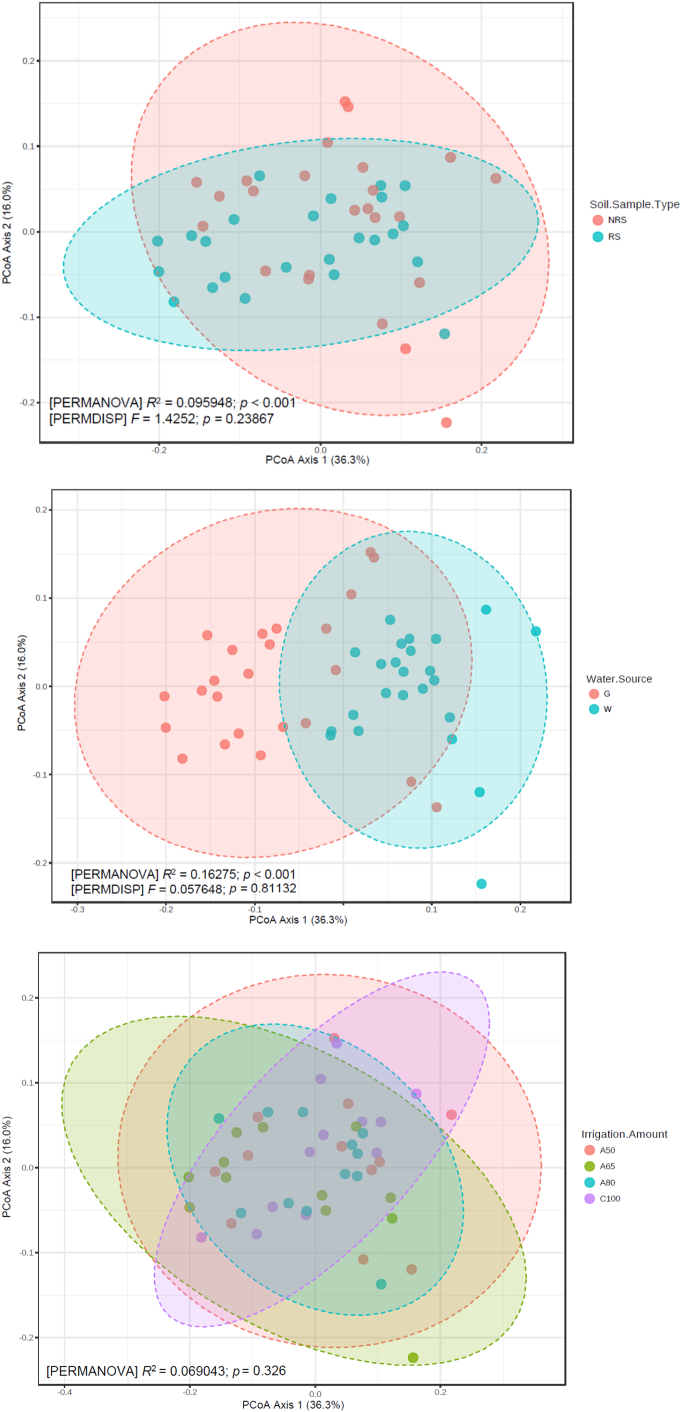
Principal Coordinate Analysis of soil bacterial communities based on weighted UniFrac distance metrics of OTUs. RS refers to rhizosphere, NRS refers to non-rhizosphere, G refers to groundwater, W refers to livestock wastewater, C refers to conventional furrow irrigation, A refers to alternate-furrow irrigation. 100, 50, 65 and 80 refer to 100%, 50%, 65% and 80% of full irrigation amount per plot, respectively. Shaded ellipses represent the 95% confidence interval surrounding the treatment centre of mass.

PCoA revealed separation of bacterial assemblages between the rhizosphere and the non-rhizosphere. Rhizosphere-associated OTUs showed a decrease in variability compared to the non-rhizosphere-associated OTUs (Fig. 3). This separation was also evident from cluster analysis (Fig. S3). Two-factor PERMANOVA (Table 2) indicated a significant divergence in soil OTUs, depending upon irrigation water source but not on irrigation rate.

**Table 2:** Two-way permutational multivariate analysis of variance of bacterial community and ARGs in rhizosphere and non-rhizosphere.

Soil compartment	Source of variation	Bacterial community			ARGs	
		F	*p*		F	*p*
Rhizosphere	Water source	13.31	<0.001		30.27	<0.001
	Irrigation amount	1.07	0.38		9.69	<0.001
	Interaction	1.01	0.41		7.16	<0.001

Non-rhizosphere	Water source	2.39	0.02		11.23	<0.001
	Irrigation amount	1.07	0.35		5.87	<0.001
	Interaction	1.05	0.38		5.99	<0.001

### 3.5 Relative abundance of ARGs and *intI1* in soil

#### 3.5.1 Correlation between relative abundance of *intI1* and ARGs

All seven ARGs were positively correlated with *intI1* (Table S4), suggesting that *intI1* may play an important role in the mobility of ARGs. The association between *sulI* and *intI1* genes was the strongest in all associations between ARGs and *intI1* studied in both the rhizosphere (*r* = 0.97, *p* < 0.001) and the non-rhizosphere (*r* = 0.68, *p* < 0.001).

#### 3.5.2 Water quality effects in rhizosphere and non-rhizosphere

**Fig. 4:**
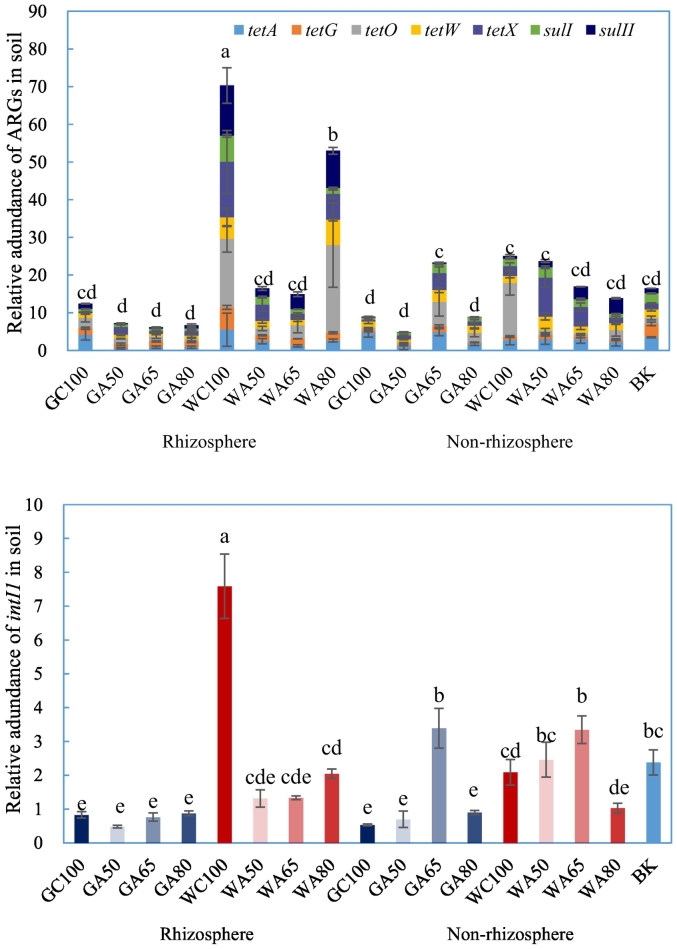
The abundance of antibiotic resistance genes and *intI1* in soil relative to the soil before fertilization and cultivation. G refers to groundwater, W refers to livestock wastewater, C refers to conventional furrow irrigation, A refers to alternate-furrow irrigation. 100, 50, 65 and 80 refer to 100%, 50%, 65% and 80% of full irrigation amount per plot, respectively. BK refers to bare plot soil with base fertilizer only but no cultivation and no irrigation. The data are expressed as the mean ± standard deviation. Lower-case letters above each column denote groups between which significant differences occur at *p* < 0.05 determined from Duncan's post-hoc pairwise comparisons.

**Fig. 5:**
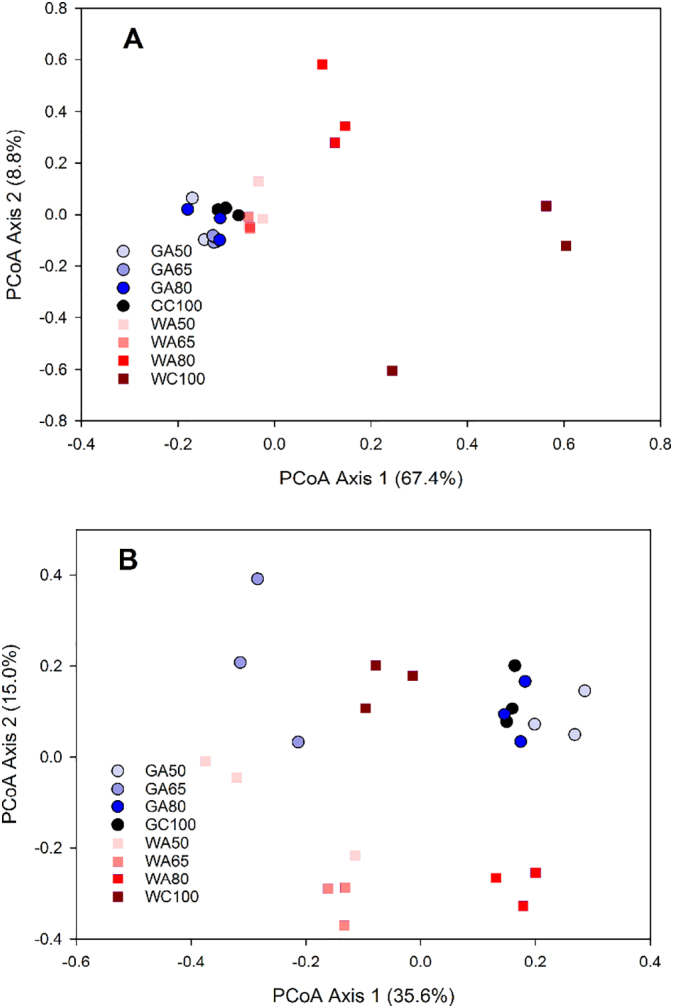
Principal Coordinate Analysis (PCoA) of antibiotic resistance and class I integron integrase genes using Gower distance metrics in rhizosphere (A) and non-rhizosphere (B). G refers to groundwater, W refers to livestock wastewater, C refers to conventional furrow irrigation, A refers to alternate-furrow irrigation. 100, 50, 65 and 80 refer to 100%, 50%, 65% and 80% of full irrigation amount per plot, respectively.

**Fig. 6:**
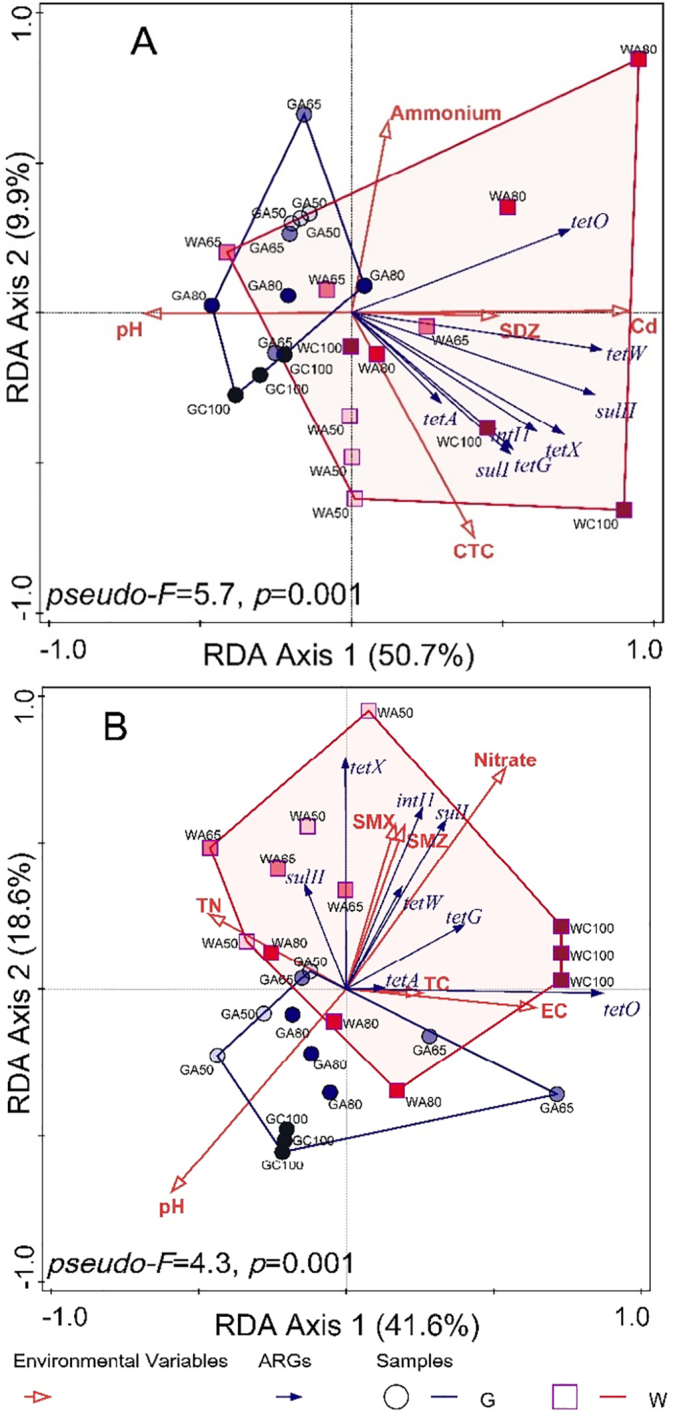
Redundancy Analysis (RDA) presenting associations of antibiotic resistance and class I integron integrase genes with measured environmental variables in rhizosphere (A) and non-rhizosphere (B). Environmental variables were selected using Forward Selection. G refers to groundwater, W refers to livestock wastewater. C refers to conventional furrow irrigation, A refers to alternate-furrow irrigation. 100, 50, 65 and 80 refer to 100%, 50%, 65% and 80% of full irrigation amount per plot, respectively. TC refers to Tetracycline, CTC refers to Chlortetracycline, SDZ refers to Sulfadiazine, SMX refers to Sulfamethoxazole, and SMZ refers to Sulfamerazine, TN to total nitrogen, EC to electrical conductivity, and Cd to available cadmium.

Relative to groundwater irrigation, wastewater irrigation significantly increased the abundance of ARGs and *intI1* in both the rhizosphere and the non-rhizosphere (Fig. 4, Table 2). Furthermore, abundance of ARGs and *intI1* between the different soils was separated in PCoA ordination (Fig. 5). In the rhizosphere, groundwater- and wastewater-irrigation treatments were separated on the first PCoA axis (associated with 67% of the variation in gene abundance). No such separation of soils based upon the abundance of ARGs was evident in the non-rhizosphere, where groundwater-irrigated and wastewater-irrigated soils were separated on the second PCoA axis (accounting for only 15% of the variation in gene abundance). There was also a clear separation of wastewater irrigation rates along the first axis. Similar overall patterns were evident in RDA, which identified strong and significant associations between ARGs, *intI1* and the increased concentrations of antibiotics in the wastewater-irrigated soils which was not evident in groundwater-irrigated soils (Fig. 6). For the rhizosphere, RDA separated the effects of irrigation water source on the first axis, representing 51% of the variability accounted for by the model. Bioavailable Cd (accounting for 23.8% of variability, *pseudo-F* = 9.2; *p* = 0.001), pH (12.1% of variability, *pseudo-F* = 3.4; *p* = 0.039) and the concentration of the sulfonamide compound SDZ (12.1% of variability, *pseudo-F* = 3.0; *p* = 0.042) were strongly associated with this separation of wastewater- and groundwater-irrigated soils. All ARGs and *intI1* showed some level of association with increased concentrations of SDZ and available Cd. None was associated with the increased pH, evident in groundwater-irrigated soils. NH_4_^+^-N (6.9% of variability, *pseudo-F* = 2.9; *p* = 0.05) and the tetracycline compound CTC (6.3% of variability, *pseudo-F* = 2.9; *p* = 0.04) were more closely associated with the second axis, suggesting a reduced association with the influence of irrigation water source and less influence upon the abundance of ARGs.

Such a clear separation between irrigation water sources in the rhizosphere was not evident in the non-rhizosphere, where groundwater and wastewater treatments were separated on the second axis, which accounted for only 19% of the variability. In these non-rhizospheric soils, EC (10.6% of variability, *pseudo-F* = 3.5; *p* = 0.02) and total nitrogen (12.0% of variability, *pseudo-F* = 3.0; *p* = 0.041) exerted the greatest influence on the primary axis (accounting for 42% of variability) while the tetracycline compound TC (10.4% of variability, *pseudo-F* = 3.1; *p* = 0.047) had the lesser influence. This separation was driven largely by the application of wastewater at the 100% rate. The genes *tetO* and *tetA* were associated with this axis, with the latter only weakly. All other genes were associated with increased concentrations of the sulfonamide compound SMX (10.0% of variability, *pseudo-F* = 2.7; *p* = 0.049), SMZ (8.3% of variability, *pseudo-F* = 3.1; *p* = 0.038), and NO_3_^−^-N (6.9% of variability, *pseudo-F* = 2.8; *p* = 0.037).

#### 3.5.3 The effects of alternate-furrow irrigation and varying irrigation amounts

Wastewater irrigation resulted in increased abundance of ARGs and *intI1* in the rhizosphere, especially at high irrigation rates. The effects of AFI and irrigation amounts were evident in PCoA (Fig. 5), and the two-factor PERMANOVA identified a significant effect of irrigation rate (Table 2). The irrigation rate effects were also evident in RDA, with a few exceptions, especially in the wastewater irrigated plots (Fig. 6).

The abundance of ARGs and *intI1* in the rhizosphere was significantly reduced by AFI compared to CFI. Such significant differences were not observed for groundwater irrigation, possibly because the abundance of ARGs was already low even at the full irrigation rate. Under AFI with wastewater, the 80% rate was associated with the highest relative abundance of ARGs and *intI1* in the rhizosphere; but there was no significant difference in the abundance of the ARGs and *intI1* between the 50% and 65% rates. Again, reducing irrigation rate did not have an impact upon the abundance of ARGs in soil under groundwater irrigation.

### 3.6 The relative abundance of ARGs and *intI1* in plant tissues

#### 3.6.1 Water quality effects

**Fig. 7:**
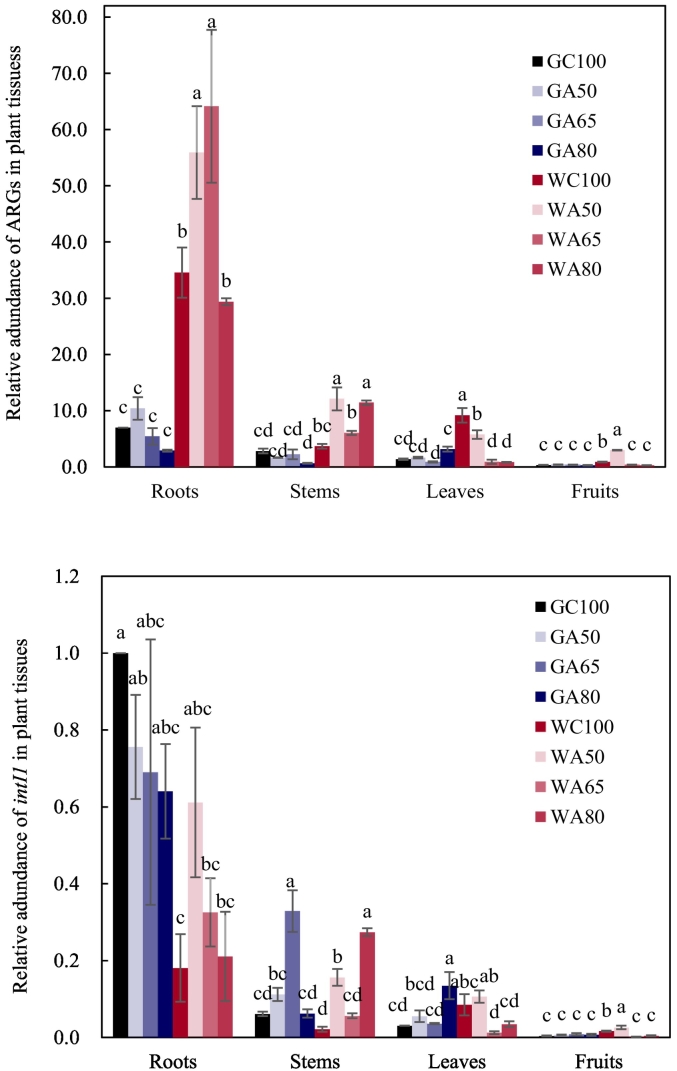
The relative abundance of antibiotic resistance and class I integron integrase genes in plant tissues. The relative abundance of antibiotic resistance genes is the sum of the relative abundance of *tetA*, *tetG*, *tetO*, *tetW*, *tetX*, *sulI* and *sulII* (Table S5). 100, 50, 65 and 80 refer to 100%, 50%, 65% and 80% of full irrigation amount per plot, respectively. The data are expressed as the mean ± standard deviation. Lower-case letters above each column denote groups between which significant differences occur at *p* < 0.05 determined from Duncan's post-hoc pairwise comparisons.

Irrigation with wastewater resulted in an increase in ARGs abundance within different plant tissues compared to groundwater irrigation, especially in the roots (Fig. 7). For *intI1*, the significant increase in gene abundance due to wastewater irrigation was only found in the stems at 80% rate as well as in the fruits at 100% and 50% rates.

#### 3.6.2 Alternate-furrow irrigation effects

Under groundwater irrigation, AFI did not influence the abundance of genes in plant tissues notably, compared to CFI. When using wastewater, AFI at 50% rate significantly decreased ARGs abundance in stems, but significantly increased ARGs abundance in roots, leaves and fruits as well as *intI1* abundance in roots, stems and fruits, compared to CFI. Hence, AFI effects upon ARGs abundance in plant tissues were not as consistent as its effects on ARGs abundance in soil.

#### 3.6.3 Irrigation amount effects under AFI

When using wastewater for irrigation, the abundance of ARGs and *intI1* in plant roots did not decrease in response to decreased irrigation rates. The abundance of ARGs in roots reached its maximal values at the 65% rate while the abundance of *intI1* in roots reached its maximum under the 50% rate. For groundwater irrigation, no significant differences in ARGs abundance were observed in roots in response to different irrigation rates. Regardless of the water source, there was no consistent increase in ARGs abundance in stem and leaf materials after increasing irrigation amount. The abundance of ARGs in fruits was the lowest in all plant tissues examined in this study. Under AFI with wastewater, the abundance of ARGs and *intI1* within the fruits at the 50% rate was significantly higher than that at the 65 and 80% rates.

### 3.7 The concentration of antibiotic compounds in plant tissues

**Fig. 8:**
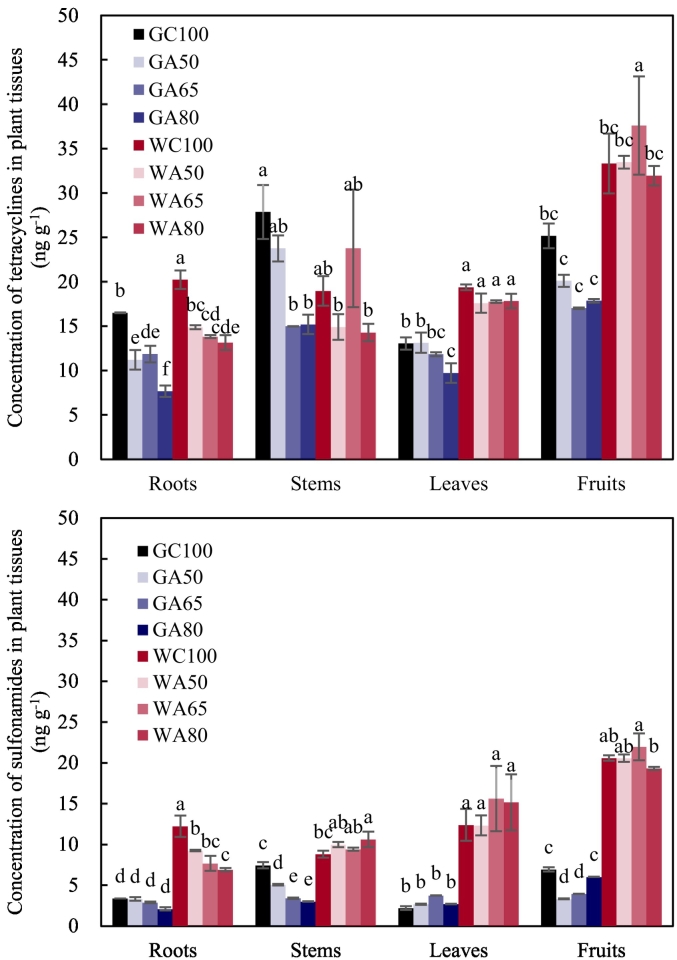
The concentration of antibiotic compounds in plant tissues. The concentration of tetracyclines is the sum of the concentrations of tetracycline, chlortetracycline and oxytetracycline (Table S6). The concentration of sulfonamides is the sum of the concentrations of sulfadiazine, sulfamethoxazole and sulfamerazine (Table S6). G refers to groundwater, W refers to livestock wastewater, C refers to conventional furrow irrigation, A refers to alternate-furrow irrigation. 100, 50, 65 and 80 refer to 100%, 50%, 65% and 80% of full irrigation amount per plot, respectively. The data are expressed as the mean ± standard deviation. Lower-case letters above each column denote groups between which significant differences occur at *p* < 0.05 determined from Duncan's post-hoc pairwise comparisons.

Antibiotics in all plant tissues were above the detection limit. Similar to that in soil, the concentrations of sulfonamide compounds in plant tissues were much lower than tetracycline compounds (Fig. 8). Wastewater irrigation resulted in a greater accumulation of antibiotics in tissues compared to groundwater irrigation with a few exceptions. Compared to CFI with wastewater, antibiotic concentrations in roots were significantly lower in AFI at the 50% irrigation rate. With groundwater irrigation, tetracycline concentrations in roots and sulfonamide concentrations in stems and fruits under AFI at the 50% rate were significantly lower than that under CFI. AFI had an apparently negative effect upon accumulation of antibiotics in plant tissues.

Under wastewater irrigation, there was no significant difference in concentration of antibiotics in roots, stems and leaves among all three AFI treatments; but antibiotic concentrations in fruits at 65% rate were significantly increased. Under groundwater irrigation, the tetracyclines in roots and leaves at 80% rate were significantly lower than that at 50% rate, and the sulfonamides in stems at 50% rate and the sulfonamides in fruits at 80% rate were both significantly higher than that at other two AFI rates. In all treatments, the concentration of sulfonamides in roots were higher than that in the associated soil.

## 4 Discussion

The source of water used in irrigation exerted significant effects on soil bacterial communities, the concentrations of antibiotic compounds, the abundance of ARGs and *intI1* in both the rhizosphere and plants. AFI reduced the abundance of ARGs in soil compared to CFI, consistent with our hypothesis. Under groundwater irrigation, different irrigation rates had no significant effect upon the abundance of ARGs in soil. Nevertheless, irrigation rate under AFI had no consistent effects upon the abundance of ARGs in plants, and nor the concentrations of antibiotic compounds in soil and plants.

### 4.1 Potential effects of horizontal genetic transfer on dispersion of ARGs

Horizontal genetic transfer plays an important role in spread of ARGs within microbial communities. Conjugation facilitated by the conjugative machinery, which is encoded either by genes on autonomously replicating plasmids or by integrative conjugative elements in the chromosome, is thought to have the greatest influence on the dissemination of ARGs (Smillie et al., 2010). In this study, we observed closely associated behaviour of the *sulI* and class 1 integron integrase genes, which was evident in both correlation analyses (Table S4) and RDA (Fig. 6). The integron integrase gene, *intI1*, is an important marker for vectors associated with the propagation of antibiotic resistance (Gillings et al., 2008). Typically, *intI1* is associated with *sul1* at the 3′-conserved region, capturing gene cassettes that confer additional and combined resistance to hosts (Heuer and Smalla, 2007). This may explain their strong association observed in the soils in our study. Such associations have been found in other studies (Du et al., 2014; Lin et al., 2016; Peng et al., 2017; Wang et al., 2014). For example, in a long-term experimental study, significant associations between ARGs and *intI1* were observed, especially between *sulI* and *intI1* (Peng et al., 2017). Additionally, conjugative machinery may enable the mobilization of plasmids that are non-conjugative, as observed, for example, with small, multi-copy, broad-host-range plasmids (Smalla et al., 2000). Plasmid groups IncP-1, IncQ, IncN and IncW are frequently detected (Binh et al., 2008) and the diversity of plasmids that transfer resistance genes has recently been reviewed (Palmer et al., 2010). Among these plasmids, the Inc18-type has been identified playing an important role in antibiotic resistance (Heuer et al., 2011). Recent studies also suggest roles for naked extracellular DNA and transduction by bacteriophages or gene transfer agents resembling bacteriophages in the horizontal genetic transfer of ARGs (von Wintersdorff et al., 2016). We also found a strong relationship between bioavailable Cd and ARGs in the rhizosphere using RDA, suggesting that increasing the concentrations of Cd was associated with increased abundance of all ARGs measured in this study. This may arise from co-selection of ARGs and metal resistance genes, which often occurs on the same mobile genetic elements (Baker-Austin et al., 2006). Although such co-selection is well established, its consequences for the management of wastewater irrigation has received little attention. Our results suggest that this lack of attention needs to be addressed, as simply limiting or removing antibiotics from farms may not be sufficient to limit the spread of ARGs in receiving soils.

### 4.2 Correlation between antibiotics and relative abundance of ARGs

Similar to their distribution in irrigation water, the concentration of sulfonamides in soil was much lower than tetracyclines. Soil is more adsorptive to tetracyclines than to sulfonamides (Hamscher et al., 2005). As a result, sulfonamides are more mobile and can be leached out of soil, whereas tetracyclines are less mobile and can accumulate in soil. When antibiotics and ARGs associated with the wastewater entered soil via irrigation, it is expected that the ARGs abundance would initially increase before attaining a plateau, followed by a falling due to degradation and leaching (Heuer et al., 2008). All ARGs studied here were positively correlated with concentrations of the two classes of antibiotic compound in the rhizosphere, while in non-rhizosphere positive correlations of ARGs were identified only for sulfonamides, especially SMX and SMZ. In non-rhizosphere, ARGs and antibiotics had no consistent associations (Table S4). In RDA, it was found that ARGs abundance was more associated with the more bioavailable sulfonamides than the less mobile tetracyclines in both rhizosphere and non-rhizosphere, revealing that antibiotic bioavailability, especially the less concentrated but more mobile sulfonamides, played an important role in spreading ARGs.

### 4.3 Water quality effects on ARGs

High ARGs abundance and antibiotic compound concentrations in soils were found after wastewater irrigation, consistent with previous reports (Ji et al., 2012; Negreanu et al., 2012). This is probably due to the high ARGs abundance and antibiotic concentrations in the wastewater. RDA provided evidence that wastewater-irrigated soils were associated with increased antibiotics, bioavailable Cd, nitrogenous compounds and high EC, whereas groundwater-irrigated soils were associated with increased pH. Under wastewater irrigation, the abundance and distribution of ARGs in rhizosphere varied with irrigation rate, while in the groundwater-irrigated soils the irrigation rates had negligible effect upon the abundance and distribution of genes, probably because of the relatively low abundance of genes in the groundwater. We also found significantly different bacterial communities in soil, dependent upon the source of irrigation water and soil compartment, but the irrigation rate did not have noticeable effects upon OTU abundance and diversity (Fig. 3).

### 4.4 Effects of AFI on the relative abundance of ARGs

AFI achieved a high fruit yield using only 50% of the amount of water used in CFI (Fig. S4). Meanwhile, AFI reduced the abundance of ARGs but not the concentration of antibiotic compounds in the rhizosphere. Despite CFI and AFI with wastewater resulting in similar antibiotic concentrations in the rhizosphere, the abundance of ARGs was consistently lower under AFI suggesting that AFI indeed reduced the mobility and bioavailability of the antibiotic compounds. Though AFI wetted only half of the roots during each irrigation, the difference in water matric potential between soils in the dry and wetted furrows could drive water to flow from the wetted half to the dry half across the root zone, increasing water use efficiency as a result (Graterol et al., 1993; Kang et al., 2000b). Root uptake actively moves distant water into the rhizosphere, but this is reduced under AFI due to the decrease in transpiration (Kang et al., 2000b). Furthermore, because most antibiotics found in our study were tetracyclines which are adsorptive to soil, root-induced water flow could only bring very limited mobile antibiotics into the rhizosphere. Combination of these physical processes rendered the antibiotics in the rhizosphere under AFI lower than that under CFI. It has been reported that AFI can maintain high bacterial biomass, even under severe water deficit irrigation (Wang et al., 2008). AFI had no significant effect on soil pH, EC, concentrations of nitrogen and heavy metals (Figs. S1 and S2). Thus, the reduced abundance of ARGs under AFI compared to CFI was likely to have resulted from the decreased bioavailability of Cd and antibiotic compounds.

Biologically, AFI - akin to partial water stress - promotes synthesis of ABA by roots in the dry soil (Kang et al., 2000b). ABA has been found capable of modulating plant-pathogen interactions apart from regulating leaf-stomatal conductance (Fan et al., 2009). The change in ABA leads to changes in other plant hormones and genes expression, which in turn might affect the diffusion of ARGs abundance in root endophytes and explain the increased abundance of ARGs in the roots under wastewater irrigation as found in this paper.

### 4.5 Effects of AFI irrigation amount on the relative abundance of ARGs

When the AFI irrigation amount with wastewater increased from 50% to 80%, ARGs abundance increased significantly in the rhizosphere. Reducing irrigation rate did not have a significant effect upon the bacterial communities in our study, possibly because of the CFI with groundwater prior to the harvest. Since ARGs abundance in groundwater was relatively low compared to that in wastewater, no significant effects of irrigation rate were observed in the groundwater treatments. In wastewater irrigation, the available Cd in the rhizosphere and the increased EC in the non-rhizosphere was associated with the irrigation rate (Fig. 6).

In wastewater-irrigated rhizosphere, antibiotic compound behaviour at the 80% rate was markedly different from that at the 50% and 65% rates. At the 80% rate, the water supply rate exceeded soil infiltration rate and surface water runoff occurred subsequently. As a result, there was no significant difference between soil water content beneath the dry and the wetted furrows (Wang et al., 2008). This might have made some microbial and plant physiological processes at the 50% and 65% rates differ from that at the 80% rate, such as root exudation and soil-plant-microbe interactions. As such, similar antibiotics contents in the rhizosphere and the non-rhizosphere were found at the 80% rate, and the concentration of antibiotics did not increase with the irrigation amounts. One possible reason is that in the 80% rate treatment, part of the irrigation water leached out of the top 0–20 cm soil where the soil samples were taken from. The main component of 0–20 cm and 20–40 cm soil in our experiment is silt and sand respectively (Du et al., 2016), and thus the irrigation water may have moved to the deeper soil. In the 50% and 65% treatments, plants were under water stress and their roots could possibly have gone deep to explore water. In contrast, in the 80% treatment, water was more available and the roots proliferated mainly in the top soil. The increased root activity in the top soil at the 80% rate might have facilitated water flow into the deep soil and degradation of the antibiotics in the rhizosphere concurrently, resulting in a decreased antibiotic concentration.

Due to the decreased antibiotic concentrations in the rhizosphere in the 80% treatment, the concentration of the antibiotics and the abundance of ARGs in plant roots were also reduced. Unlike the water quality effects, the irrigation-rate effects were mainly associated with ARGs, heavy metals, nutrients, total concentration of antibiotics and their bioavailability.

### 4.6 Plant effects on antibiotics and relative abundance of ARGs

Plant effects include roots and canopies. They interact via complicated physical and biochemical processes, depending on plant species, soil types, climate and land management (Waring et al., 2015). Canopies control soil moisture and temperature, as well as gaseous flows into and out of soil, thereby soil microbial activities and biochemical processes. Root uptake of water can convectively bring distant soluble chemicals such as heavy metals, antibiotics and ARGs into the rhizosphere, and subsequently accumulate those that are taken up by the roots (Huang et al., 2015; Liu et al., 2016) especially when the plants act as hyperaccumulators (Liu et al., 2013). Our study found accumulation of antibiotics and ARGs in the plants, consistent with previous studies (Wu et al., 2013; Ye et al., 2016). For the pepper variety of Fulong F1 used in our study, the antibiotic concentrations in the leaves and fruits were higher than in the roots, as opposed to the Anaheim chili pepper in the study of Wu et al. (2013) and the bell pepper in the study of Ye et al. (2016). The antibiotics concentrations in the pepper roots reported in Wu et al. (2013) were comparable with that in our study, while the antibiotics concentrations in the roots reported in Ye et al. (2016) were up to 100 times higher than that in our study. For SMX, both Wu et al. (2013) and our studies found that its concentrations in roots were higher than that in the growing media. For SDZ examined by us and Ye et al. (2016), we both identified that its concentrations in the roots were higher than that in the growing media. Ye et al. (2016) observed that the TC concentrations in the roots were higher than that in the growing medium, in contrast to parts of our results probably because the agronomical managements and medium types made bioavailability of the antibiotics in our study differ from theirs.

A variety of crops have been found capable of taking up antibiotics from soil, including lettuce, common barley and cucumber (Lillenberg et al., 2010), ryegrass (Winker et al., 2010), spinach (Wu et al., 2013), potato (Dolliver et al., 2007), common bean and radish (Migliore et al., 2003), green onion and cabbage corn, (Kumar et al., 2005), rice (Boonsaner and Hawker, 2012), soybean (Boonsaner and Hawker, 2010), and others (Jiao et al., 2018). For antibiotics that accumulate in roots, high antibiotic residues are expected to be found in tuber crops, while for antibiotics with high translocation potential they might end up in leaves and fruits. Antibiotics with high translocation potential should receive more attention especially for leafy and fruit vegetables such as tomato, pepper, and cucumber, among other.

Antibiotics enter plant tissues through root uptake and the rhizosphere effect is hence an important concern. Plant species differ in their rhizosphere effects due to their difference in root exudation, nutrient absorption and root architecture. Wetting-drying induced by alternate-furrow irrigation in our experiment impacted root architecture, thereby influencing the antibiotics and ARGs in both the rhizosphere and the non-rhizosphere. In addition to root exudations, imbalance in root uptake of anions and cations, as well as the change in soil structure induced by root activity would alter soil pH and other biochemical processes. All these would change the bioavailability of metals and organic pollutants (Sabir et al., 2014), thereby indirectly influencing the adsorption/desorption and bioavailability of antibiotics and the dispersion of ARGs.

Here, we compared the rhizosphere effects on ARGs between the rhizosphere and the non-rhizosphere. At high wastewater irrigation rates (100% and 80%), all ARGs were more abundant in the rhizosphere due to either a wider spread of genes through the rhizosphere bacterial community or the greater abundance of the ARGs-harbouring cells in rhizosphere. At low irrigation rates (50% and 65%), there was no significant difference in ARGs abundance. ARGs behaved differently in response to different environmental factors and different soil compartments (Fig. 5, Fig. 6). The rhizosphere was more responsive to water quality than the non-rhizosphere. In the rhizosphere, the behavior of ARGs was influenced by available Cd and SDZ. ARGs abundance was also slightly influenced by CTC, possibly because of the increased bioavailability resulting from the high organic matter in the rhizosphere (Hung et al., 2009). Similarly, in non-rhizosphere, genes were influenced by the more mobile SMX and SMZ compounds than the less mobile TC. Antibiotic bioavailability and mobility in soil proved to play a key role in ARGs distribution.

Our recent results from rhizobox experiments filled with the same soil have shown that repeated irrigation with wastewater rendered the ARGs abundance in the rhizosphere higher than that in non-rhizosphere after 30 days and 60 days of irrigation (Cui et al., 2018), and it was likely that in our study, ARGs abundance in the rhizosphere was also higher before the CFI with groundwater was restored. In our experiment, there was a gap of almost 60 days between the last wastewater irrigation and harvest. ARGs abundance may have attenuated during this period, and the differences in their abundances between the rhizosphere and the non-rhizosphere were likely to have changed. At high irrigation rates, the soil water content was relatively high and the weakly-adsorbed sulfonamides were relatively easy to move into the rhizosphere driven by root-induced water movement. Thus, increasing irrigation rate was likely to have delivered more antibiotic compounds into the soil, some of which moved into the rhizosphere even after the wastewater irrigation ceased. For antibiotics that roots do not actively take up, they accumulated in the rhizosphere enhancing spread of ARGs between organisms compared to that in the non-rhizosphere. At low irrigation rates, the availability of antibiotic compounds was low and the soil was more aerobic. Such conditions might make microbes capable of producing enzymes to degrade antibiotic compounds less competitive. This, along with ARGs attenuation, could reduce the ARGs abundance in the rhizosphere.

### 4.7 Potential application

Increasing water use efficiency and safely reusing recycled wastewater are essential in developing sustainable agriculture not only for China but also for countries that face water resources scarcity. In addition to improving agronomical management, efficient irrigation methods such as AFI would play an important role in achieving this target as demonstrated in research across the globe (Abd El-Halim, 2013; Aujla et al., 2007; Bogale et al., 2016; Du et al., 2013; Graterol et al., 1993; Grimes et al., 1968; Ramalan and Nwokeocha, 2000; Samadi and Sepaskhah, 1984; Sepaskhah and Hosseini, 2008; Sepaskhah and Khajehabdollahi, 2005; Siyal et al., 2016; Webber et al., 2006; Zhang et al., 2012). We took pepper as the model plant in this work, but the method is applicable to other crops (Kang and Zhang, 2004). Also, the Calcaric fluvic cambisol investigated in this study is a type of soil existing widely in both arid and semi-arid areas in the world (Dabach et al., 2016; Márquez et al., 2017; Molina et al., 2016; Parihar et al., 2016; Schirrmann et al., 2016; Sharma et al., 2017). Our results about the feasibility of using AFI to control spread of ARGs in soil-plant systems has potential implications in these regions. In terms of controlling relative abundance of ARGs and antibiotics concentration in soil and plants, AFI at the 80% rate appeared to be most efficient in our experiment, but this is likely to vary with soil and crops and further research is required. As a first attempt, we did show that alternately wetting and drying soil via alternate-furrow-irrigation could control the spread of ARGs into plant issues through modulating rhizosphere processes.

## 5 Conclusions

We studied the differences in ARGs distribution in soil and plant tissues between conventional furrow irrigation (CFI) and alternate-furrow irrigation (AFI) with groundwater and wastewater at different irrigation rates. ARGs abundance in the rhizosphere was more sensitive to wastewater-irrigation than in the non-rhizosphere. Compared with CFI, AFI reduced ARGs abundance in the rhizosphere, but could risk the occurrence of ARGs in plant tissues. Water quality had a manifest effect on the spread of ARGs: the genes were more responsive to wastewater irrigation than to groundwater irrigation. Under AFI with wastewater, decreasing the irrigation amount could reduce the ARGs abundance in the rhizosphere, but not the ARGs accumulation in plant tissues. Antibiotic bioavailability was of great significance in dispersion of ARGs. Further research is hence required to achieve water savings without a risk to public health arising from dissemination of antibiotic resistance in microorganisms when using livestock wastewater for irrigation.

We measured the properties of soil and crop only at the harvest and did not assess the availability of the antibiotic compounds in the soil. However, the effects of AFI with different irrigation rates on ARGs abundance in soil and plants were evident, and the quality of soil and fruits at the harvest is at the main public concern. We conjectured from our findings that some microbial and plant physiological processes unique to roots induced by water stress might play an important role in ARGs diffusion in the rhizosphere, which needs further research.

## Appendix A Supplementary data

Supplementary material Image 1
