# Supplementary material for: Reducing water use by alternate-furrow irrigation with livestock wastewater reduces antibiotic resistance gene abundance in the rhizosphere but not in the non-rhizosphere
Source: Sci Total Environ. 2019 Jan 15;648:12–24. doi: 10.1016/j.scitotenv.2018.08.101 (PMC6234105; doi:10.1016/j.scitotenv.2018.08.101)
Supplement: Supplementary file 1 — Supplementary material [file mmc1.docx]

**Supplementary Information**

**Reducing water use by alternate-furrow irrigation with livestock wastewater reduces antibiotic resistance gene abundance in the rhizosphere but not in the non-rhizosphere**

Yuan Liu^a^, Erping Cui^a^, Andrew L. Neal^b^, Xiaoxian Zhang^b^, Zhongyang Li^a,*^, Yatao Xiao^a^, Zhenjie Du^a^, Feng Gao^a^, Xiangyang Fan^a^, Chao Hu^a^

^a^ Farmland Irrigation Research Institute, Chinese Academy of Agricultural Sciences, Xinxiang 453002, China

^b^ Department of Sustainable Agriculture Sciences, Rothamsted Research, Harpenden, Hertfordshire AL5 2JQ, UK

*Corresponding author. E-mail address: lizhongyang1980@163.com.

*1. Measurement of soil chemical properties*

Soil pH was measured according to the national environmental protection protocol NY/T 1377-2007 (Jin et al., 2018) using a PHS-3C pH meter (Shanghai Leici, China). Soil electrical conductivity (EC) was determined according to the standard HJ802-2016 (National environmental protection standards of the PRC, 2016) by conductivity meter DDS-307 (Shanghai Leici, China). Soil organic matter (OM) was analyzed by NY/T 1121.6-2006 (Jin et al., 2018). Soil total N (TN) was analyzed by NY/T 1121.24-2012 (Jin et al., 2018) with a Kjeldahl Analyzer KDN-08A (Shanghai Hongji, China). Soil nitrate nitrogen and ammonium nitrogen were extracted according to the protocols LY/T 1228-2015 (Zuo et al., 2018) and NY/T 1848-2010 (Standards of agricultural industry of the PRC, 2010), and then were determined by UV-5500(PC) UV/VIS spectrophotometer (Shanghai Yuanxi, China). Soil available potassium was extracted by ammonium acetate, and determined by ICP-OES iCAP7400 (ThermoFisher, USA). Soil available phosphorous was determined according to NY/T 1121.7-2014 (Wang et al., 2018) with UV-5500(PC) UV/VIS spectrophotometer. Soil total Cu, Zn, Pb and Cd was extracted by HNO_3_-HF-HClO_4_, and analyzed with ICP-MS iCAP Qc (ThermoFisher, USA). Soil available Cu, Zn, Pb, Cd was determined according to HJ 804-2016 (Jiang and Zhou, 2018), and measured with ICP-OES iCAP7400.

*2. Antibiotic compounds analysis*

Water samples (10 mL) were filtered through 0.45 μm glass fiber filters and 0.80 g L^-1^ Na_2_EDTA was added to the samples and allowed it to react for 1 h. Then, 0.1 M HCl or NaOH was used to adjust the pH of samples to 5.0. Oasis HLB plates (60 mg, Waters, USA) were successively activated with 2.0 mL methanol, 2.0 mL ultra-pure water and 1.0 mL ultra-pure water (pH 5.0 ± 0.2). Samples were passed through the plates at a rate of 0.4 mL min^-1^. The plates were rinsed with 2 mL ultra-pure water and dried under nitrogen gas for 30 min. Once dried, the plates were eluted with 2 mL of a mixture of methanol : acetonitrile (1:1, v/v). The eluates were dried under gentle nitrogen gas at 40℃ and later diluted to a volume of 100 μL with methanol : water (1:1, v/v). Finally, the treated samples were analyzed by Ultra-high Performance Liquid Chromatography tandem Mass Spectrometry (UPLC-MS/MS).

Freeze-dried solid samples (75 mg) were extracted in 3 mL of a mixed solution of 1.5 mL methanol and 1.5 mL Na_2_EDTA-McIlvaine with ultrasonication (50 kHz) for 10 min, then centrifuged at 3,000 rpm for 20 min. The procedure repeated three times and the supernatants collected after each step were pooled. 1 mL of each supernatant mixture was diluted to 10 mL with ultra-pure water. The same process was followed with the liquid samples.

The final extracts were analyzed by a UPLC-MS/MS system equipped with an Agilent 1290 Infinity UHPLC and an Agilent 6470 Triple Quadruple MS/MS (Agilent Technologies, USA). All target antibiotics were separated on an XSelect HSS T3 Column (2.5 μm, 2.1 × 100 mm, Waters Co., Massachusetts, USA) and identified and quantified by MS/MS in multi-reaction monitoring (MRM) mode. MS/MS analysis was performed in the positive electrospray ionization (ESI) mode. The specific instrument conditions of the six compounds are summarized in Table S1. The determination was conducted by Shanghai Personal Biotechnology Co., Ltd. (Shanghai, China).

*3. MiSeq pyrosequencing*

PCR amplification of the bacterial 16S rRNA gene V3–V4 variable region was performed using the forward primer 5’-ACTCCTACGGGAGGCAGCAG-3’ (338F) and the reverse primer 5’-GGACTACHVGGGTWTCTAAT-3’ (806R) (Xu et al., 2016). The reaction mixture and thermal profile of the PCR amplifications were according to Huang et al. (2016). After the PCR products were purified, they were adjusted to equal quantities, and paired-end 2×300 base pair (bp) sequencing was performed on an Illumina MiSeq sequencing platform by Shanghai Personal Biotechnology Co., Ltd. (Shanghai, China).

Sequences were examined for quality using the default arguments in the split_libraries python script with the exception of increasing primer mismatch from 0 to 2, and then assigned to each sample based on unique 10-bp barcodes. After removing barcode and primer sequences, the sequences were clustered into operational taxonomic units (OTUs) at a level of 97% sequence similarity and annotated using BLAST searches against the Greengenes (Release 13.8, http://greengenes.secondgenome.com/, bacteria) database using the Quantitative Insights into Microbial Ecology (QIIME) software package version 1.8.0 (Caporaso et al., 2010).

*4. Relative quantification of ARGs and* intIl

All qPCR reactions were repeated three times. The primer description can be found in Table S2 of the Supporting Information. All qPCR reactions were performed using CFX-96 touch real-time PCR detection system (Bio-Rad, USA). Cycle conditions were: 95 °C for 5 minutes, followed by 45 cycles of 95 °C for 15 s, 60 °C for 30 s and 72 °C for 30 s. A threshold cycle (C_t_) of 36 was used as the detection limit (Malvick and Impullitti, 2007). Generally, the technical triplicates were tested during separate testing occasions (plate and day of testing) as a method of quality control. The 2^-ΔΔCt^ method (Livak and Schmittgen, 2001; Zhu et al., 2013) was used to compare relative abundance between samples:

ΔC_t_ = C_t,(ARG or_ *_intI1_*_)_ – C_t,(16S)_

ΔΔC_t_ = ΔC_t,(Target)_ – ΔC_t,(Ref)_

where C_t_ is the threshold cycle, ARG is one of the antibiotic resistance gene assays, *intI1* is the *intI1* gene assay, 16S is the 16S rRNA gene assay, Target is the experimental sample, and Ref is the reference sample. The reference sample used for comparison depended on the purpose of the analysis. When comparing differences of ARGs abundance between groundwater and wastewater, the groundwater was selected as the reference sample. When the purpose was to reveal the changes of ARGs abundance in different soils among all treatments, the original soil before cultivation and fertilization was selected as the reference sample for all the soil samples. When the purpose was to reveal the changes of ARGs abundance in different plant tissues among all treatments, the root of GC100 was selected as the reference sample.

**References**

Caporaso, J.G., Kuczynski, J., Stombaugh, J., Bittinger, K., Bushman, F.D., Costello, E.K., Fierer, N., Pẽa, A.G., Goodrich, J.K., Gordon, J.I., Huttley, G.A., Kelley, S.T., Knights, D., Koenig, J.E., Ley, R.E., Lozupone, C.A., McDonald, D., Muegge, B.D., Pirrung, M., Reeder, J., Sevinsky, J.R., Turnbaugh, P.J., Walters, W.A., Widmann, J., Yatsunenko, T., Zaneveld, J., Knight, R., 2010. QIIME allows analysis of high-throughput community sequencing data. Nat. Methods. https://doi.org/10.1038/nmeth.f.303

Huang, X., Liu, L., Wen, T., Zhang, J., Wang, F., Cai, Z., 2016. Changes in the soil microbial community after reductive soil disinfestation and cucumber seedling cultivation. Appl. Microbiol. Biotechnol. 100, 5581–5593. https://doi.org/10.1007/s00253-016-7362-6

Jiang, K., Zhou, K., 2018. Chemical immobilization of lead, cadmium, and arsenic in a smelter-contaminated soil using 2,4,6-trimercaptotriazine, trisodium salt, nonahydrate and ferric sulfate. J. Soils Sediments 18, 1060–1065. https://doi.org/10.1007/s11368-017-1822-1

Jin, C., Nan, Z., Wang, H., Li, X., Zhou, J., Yao, X., Jin, P., 2018. Effect of Cd stress on the bioavailability of Cd and other mineral nutrition elements in broad bean grown in a loess subsoil amended with municipal sludge compost. Environ. Sci. Pollut. Res. 25, 7418–7432. https://doi.org/10.1007/s11356-017-0994-y

Livak, K.J., Schmittgen, T.D., 2001. Analysis of Relative Gene Expression Data Using Real-Time Quantitative PCR and the 2^−ΔΔCT^ Method. Methods 25, 402–408. https://doi.org/https://doi.org/10.1006/meth.2001.1262

Malvick, D.K., Impullitti, A.E., 2007. Detection and quantification of *Phialophora gregata* in soybean and soil samples with a quantitative, real-time PCR assay. Plant Dis. 91, 736–742. https://doi.org/10.1094/PDIS-91-6-0736

National environmental protection standards of the People's Republic of China, 2016. Soil quality—Determination of conductivity- Electrode method, http://www.doc88.com/p-3025282450539.html

Standards of agricultural industry of the People's Republic of China, 2010. Method for determination of ammonium nitrogen, available phosphorus and rapidly-available potassium in neutrality or calcareous soil Universal extract-colorimetric method, http://www.doc88.com/p-7816449638897.html

Wang, Y., Zhang, T. an, Lyu, G., Guo, F., Zhang, W., Zhang, Y., 2018. Recovery of alkali and alumina from bauxite residue (red mud) and complete reuse of the treated residue. J. Clean. Prod. 188, 456–465. https://doi.org/10.1016/j.jclepro.2018.04.009

Xu, N., Tan, G., Wang, H., Gai, X., 2016. Effect of biochar additions to soil on nitrogen leaching, microbial biomass and bacterial community structure. Eur. J. Soil Biol. 74, 1–8. https://doi.org/10.1016/j.ejsobi.2016.02.004

Zhu, Y.-G., Johnson, T. a, Su, J.-Q., Qiao, M., Guo, G.-X., Stedtfeld, R.D., Hashsham, S. a, Tiedje, J.M., 2013. Diverse and abundant antibiotic resistance genes in Chinese swine farms. Proc. Natl. Acad. Sci. U. S. A. 110, 3435–40. https://doi.org/10.1073/pnas.1222743110

Zuo, L., Yang, R., Zhen, Z., Liu, J., Huang, L., Yang, M., 2018. A 5-year field study showed no apparent effect of the Bt transgenic 741 poplar on the arthropod community and soil bacterial diversity. Sci. Rep. 8. https://doi.org/10.1038/s41598-018-20322-3

Figure captions

Figure S1. Concentrations of available heavy metals under different irrigation regimes.

Figure S2. Soil pH, electrical conductivity (EC), organic matter, total nitrogen, nitrate nitrogen and ammonium nitrogen under different irrigation regimes.

Figure S3. Heatmap and hierarchical bi-clustering of OTU abundance in differently irrigated soils.

Figure S4. Pepper (*Capsicum annuum* L*.*) yield under different irrigation regimes.

Tables

Table S1. Operating parameters for the detection of selected antibiotic compounds in UPLC-MS/MS.

Table S2 Target Genes, primer sequence and amplicon size for ARGs, intI1 and 16S rRNA gene amplification.

Table S3 Concentration of antibiotic compounds in soil (ng g^-1^) under different irrigation regimes.

Table S4. Pearson correlation coefficients between soil antibiotics concentrations and soil gene abundance as well as coefficients between soil ARGs and *intI1* abundance.

Table S5. The relative abundance of antibiotic resistance genes in plant materials.

Table S6 Concentration of antibiotic compounds (ng g^-1^) in pepper plants grown under different irrigation regimes.

**Fig. S1. Concentrations of available heavy metals under different irrigation regimes.** G refers to groundwater, W refers to livestock wastewater, C refers to conventional furrow irrigation, A refers to alternate-furrow irrigation. 100, 50, 65 and 80 refer to 100%, 50%, 65% and 80% of full irrigation amount per plot, respectively. BC refers to the original soil before fertilization and cultivation, BK refers to bare plot soil with base fertilizer only but no cultivation and no irrigation. RS and NRS refer to rhizosphere and non-rhizosphere. The data are expressed as the mean ± standard deviation. Different lower case letters above the columns represent significant difference between treatments at *p* < 0.05 determined using Duncan’s *post* *hoc* pairwise comparisons of means.

**Fig. S2. Soil pH, electrical conductivity (EC), organic matter, total nitrogen, nitrate nitrogen and ammonium nitrogen under different irrigation regimes.** G refers to groundwater, W refers to livestock wastewater, C refers to conventional furrow irrigation, A refers to alternate-furrow irrigation. 100, 50, 65 and 80 refer to 100%, 50%, 65% and 80% of full irrigation amount per plot, respectively. BC refers to the original soil before fertilization and cultivation, BK refers to bare plot soil with base fertilizer only but no cultivation and no irrigation. RS and NRS refer to rhizosphere and non-rhizosphere. The data are expressed as the mean ± standard deviation. Different lower case letters above the columns represent significant difference between treatments at *p* < 0.05.


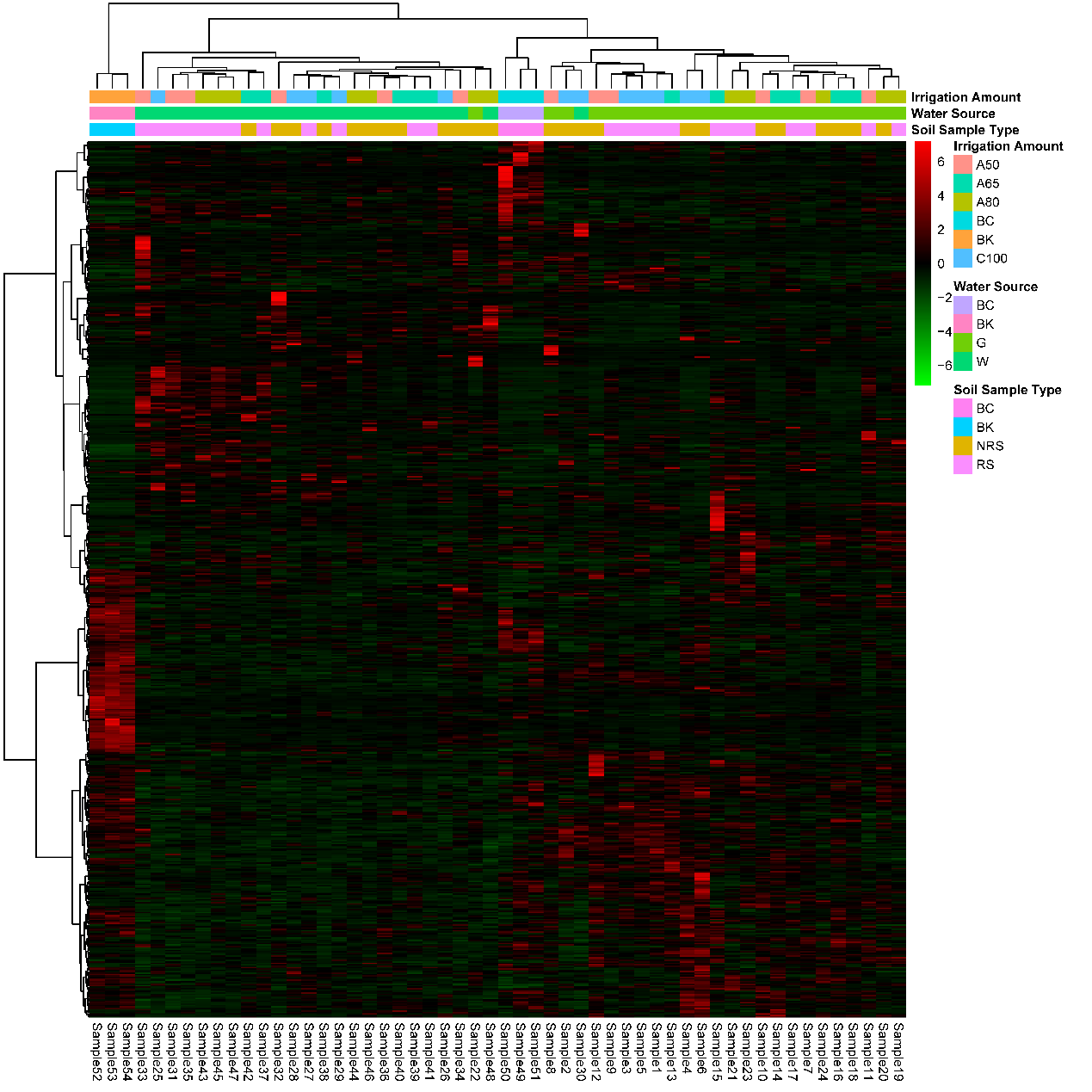


**Fig. S3. Heatmap and hierarchical bi-clustering of OTU abundance in differently irrigated soils.** G refers to groundwater, W refers to livestock wastewater, C refers to conventional furrow irrigation, A refers to alternate-furrow irrigation. 100, 50, 65 and 80 refer to 100%, 50%, 65% and 80% of full irrigation amount per plot, respectively. BC refers to the original soil before fertilization and cultivation, BK refers to bare plot soil with base fertilizer only but no cultivation and no irrigation. RS and NRS refer to rhizosphere and non-rhizosphere.

**Fig. S4. Pepper (*Capsicum annuum* L.) yield under different irrigation regimes.** G refers to groundwater, W refers to livestock wastewater, C refers to conventional furrow irrigation, A refers to alternate-furrow irrigation. 100, 50, 65 and 80 refer to 100%, 50%, 65% and 80% of full irrigation amount per plot, respectively. The data are expressed as the mean ± standard deviation. Different lower case letters above the columns represent significant difference between treatments at *p* < 0.05.

**Table S1. Operating parameters for the detection of selected antibiotic compounds in UPLC-MS/MS.**

| Compound | Mode | Precursor ion （*m*/*z*) | Product ion (*m*/*z*) | Fragmentor (V) | CE (V) |
| --- | --- | --- | --- | --- | --- |
| Oxytetracycline | + | 461.2 | 426 | 117 | 20 |
| Chlortetracycline | + | 479.2 | 444.2 | 120 | 20 |
| Tetracycline | + | 445.2 | 410.1 | 110 | 20 |
| Sulfamethoxazole | + | 254 | 92 | 115 | 30 |
| Sulfadiazine | + | 251 | 156 | 105 | 15 |
| Sulfamerazine | + | 265.1 | 92 | 100 | 33 |

**Table S2. Target Genes, primer sequence and amplicon size for ARGs, *intI1* and 16S rRNA gene amplification.**

| Target genes | Primer | Sequence (5’-3’) | Amplicon size  (bp) | Reference |
| --- | --- | --- | --- | --- |
| *tetA* | *tetA*-F | GCTACATCCTGCTTGCCTTC | 210 | (Faldynova et al., 2003) |
|  | *tetA*-R | CATAGATCGCCGTGAAGAGG |  |  |
| *tetG* | *tetG*-F | GCAGAGCAGGTCGCTGG | 134 | (Zhang et al., 2016) |
|  | *tetG*-R | CCYGCAAGAGAAGCCAGAAG |  |  |
| *tetO* | *tetO*-F | ACGGARAGTTTATTGTATACC | 171 | (Aminov et al., 2001) |
|  | *tetO*-R | TGGCGTATCTATAATGTTGAC |  |  |
| *tetW* | *tetW*-F | GAGAGCCTGCTATATGCCAGC | 168 | (Aminov et al., 2001) |
|  | *tetW*-R | GGGCGTATCCACAATGTTAAC |  |  |
| *tetX* | *tetX*-F | AGCCTTACCAATGGGTGTAAA | 278 | (Lapara et al., 2011) |
|  | *tetX*-R | TTCTTACCTTGGACATCCCG |  |  |
| *sulI* | *sulI*-F | CGCACCGGAAACATCGCTGCAC | 163 | (Zhang et al., 2016) |
|  | *sulI*-R | TGAAGTTCCGCCGCAAGGCTCG |  |  |
| *sulII* | *sulII*-F | CTCCGATGGAGGCCGGTAT | 190 | (Lu et al., 2015) |
|  | *sulII*-R | GGGAATGCCATCTGCCTTGA |  |  |
| *intI1* | *intI1*-F | CCTCCCGCACGATGATC | 280 | (Lapara et al., 2011) |
|  | *intI1*-R | TCCACGCATCGTCAGGC |  |  |
| 16S rRNA | 1369F | CGGTGAATACGTTCYCGG | 143 | (Suzuki et al., 2000) |
|  | 1492R | GGWTACCTTGTTACGACTT |  |  |

Aminov, R.I., Garrigues-Jeanjean, N., Mackie, R.I., 2001. Molecular ecology of tetracycline resistance: Development and validation of primers for detection of tetracycline resistance genes encoding ribosomal protection proteins. Appl. Environ. Microbiol. 67, 22–32. https://doi.org/10.1128/AEM.67.1.22-32.2001

Faldynova, M., Pravcova, M., Sisak, F., Havlickova, H., Kolackova, I., Cizek, A., Karpiskova, R., Rychlik, I., 2003. Evolution of antibiotic resistance in Salmonella enterica serovar Typhimurium strains isolated in the Czech Republic between 1984 and 2002. Antimicrob. Agents Chemother. 47, 2002–2005. https://doi.org/10.1128/AAC.47.6.2002-2005.2003

Lapara, T.M., Burch, T.R., McNamara, P.J., Tan, D.T., Yan, M., Eichmiller, J.J., 2011. Tertiary-Treated Municipal Wastewater is a Significant Point-Source of Antibiotic Resistance Genes into Duluth-Superior Harbor. Environ. Sci. Technol. 45, 9543–9549. https://doi.org/10.1021/es202775r

Lu, Z., Na, G., Gao, H., Wang, L., Bao, C., Yao, Z., 2015. Fate of sulfonamide resistance genes in estuary environment and effect of anthropogenic activities. Sci. Total Environ. 527–528, 429–438. https://doi.org/10.1016/j.scitotenv.2015.04.101

Suzuki, M.T., Taylor, L.T., DeLong, E.F., 2000. Quantitative analysis of small-subunit rRNA genes in mixed microbial populations via 5’-nuclease assays. Appl. Environ. Microbiol. 66, 4605–4614. https://doi.org/10.1128/AEM.66.11.4605-4614.2000

Zhang, J., Chen, M., Sui, Q., Wang, R., Tong, J., Wei, Y., 2016. Fate of antibiotic resistance genes and its drivers during anaerobic co-digestion of food waste and sewage sludge based on microwave pretreatment. Bioresour. Technol. 217, 28–36. https://doi.org/10.1016/j.biortech.2016.02.140

**Table S3. Concentration of antibiotic compounds in soil (ng g^-1^) under different irrigation regimes.** G refers to groundwater, W refers to livestock wastewater, C refers to conventional furrow irrigation, A refers to alternate-furrow irrigation. 100, 50, 65 and 80 refer to 100%, 50%, 65% and 80% of full irrigation amount per plot, respectively. BC refers to the original soil before fertilization and cultivation, BK refers to bare plot soil with base fertilizer only but no cultivation and no irrigation. RS and NRS refer to rhizosphere and non-rhizosphere. TC refers to tetracycline, CTC refers to chlortetracycline, OTC refers to oxytetracycline, SDZ refers to sulfadiazine, SMX refers to sulfamethoxazole, and SMZ refers to sulfamerazine.

|  |  | CTC | TC | OTC | SMX | SMZ | SDZ |
| --- | --- | --- | --- | --- | --- | --- | --- |
| GC100 | RS | 5.79 | 6.84 | 4.98 | 0.23 | 0.51 | 0.31 |
|  | NRS | 4.33 | 3.25 | 3.29 | 0.60 | 0.95 | 0.54 |
| GA50 | RS | 3.47 | 2.35 | 2.14 | 0.48 | 0.66 | 0.45 |
|  | NRS | 2.81 | 2.10 | 1.99 | 0.41 | 0.73 | 0.49 |
| GA65 | RS | 2.40 | 1.85 | 2.16 | 0.50 | 0.79 | 0.50 |
|  | NRS | 2.80 | 2.10 | 2.33 | 0.36 | 0.71 | 0.46 |
| GA80 | RS | 2.11 | 1.70 | 1.97 | 0.31 | 0.44 | 0.38 |
|  | NRS | 2.03 | 1.84 | 2.34 | 0.17 | 0.47 | 0.28 |
| WC100 | RS | 8.18 | 5.73 | 9.03 | 0.81 | 1.56 | 0.60 |
|  | NRS | 4.85 | 3.83 | 3.55 | 0.88 | 1.61 | 0.60 |
| WA50 | RS | 8.97 | 5.72 | 10.11 | 0.84 | 1.63 | 0.57 |
|  | NRS | 3.45 | 3.05 | 2.80 | 0.88 | 1.66 | 0.60 |
| WA65 | RS | 4.43 | 9.63 | 9.04 | 1.07 | 1.57 | 0.59 |
|  | NRS | 2.34 | 2.73 | 2.37 | 0.85 | 1.52 | 0.57 |
| WA80 | RS | 5.05 | 3.41 | 4.68 | 0.81 | 1.14 | 0.54 |
|  | NRS | 4.17 | 4.59 | 4.66 | 0.79 | 1.63 | 0.58 |
| BC |  | 1.88 | 2.43 | 2.09 | 0.79 | 1.37 | 0.54 |
| BK |  | 2.09 | 2.26 | 2.15 | 0.20 | 0.49 | 0.25 |

**Table S4. Pearson correlation coefficients between soil antibiotics concentrations and soil gene abundance as well as coefficients between soil ARGs and *intI1* abundance.** * refers to correlation is significant at the 0.05 level (2-tailed). TC refers to tetracycline, CTC refers to chlortetracycline, OTC refers to oxytetracycline, SDZ refers to sulfadiazine, SMX refers to sulfamethoxazole, and SMZ refers to sulfamerazine.

|  |  | *tetA* | *tetG* | *tetO* | *tetW* | *tetX* | *sulI* | *sulII* | *intI1* |
| --- | --- | --- | --- | --- | --- | --- | --- | --- | --- |
| Rhizosphere | SMX | 0.062 | 0.320 | 0.341 | 0.353 | 0.315 | 0.397 | 0.443^*^ | 0.346 |
|  | SMZ | 0.189 | 0.443^*^ | 0.338 | 0.346 | 0.330 | 0.576^**^ | 0.458^*^ | 0.489^*^ |
|  | SDZ | 0.068 | 0.401 | 0.347 | 0.312 | 0.290 | 0.516^**^ | 0.421^*^ | 0.447^*^ |
|  | TC | 0.197 | 0.250 | 0.045 | 0.034 | 0.119 | 0.210 | 0.176 | 0.179 |
|  | OTC | 0.248 | 0.439^*^ | 0.012 | 0.289 | 0.380 | 0.491^*^ | 0.439^*^ | 0.430^*^ |
|  | CTC | 0.379 | 0.545^**^ | 0.085 | 0.439^*^ | 0.573^**^ | 0.604^**^ | 0.524^**^ | 0.531^**^ |
|  | *intI1* | 0.563^**^ | 0.946^**^ | 0.497^*^ | 0.682^**^ | 0.702^**^ | 0.971^**^ | 0.754^**^ |  |
| Non-rhizosphere | SMX | 0.055 | 0.071 | 0.145 | 0.175 | 0.421^*^ | 0.351 | 0.620^**^ | 0.288 |
|  | SMZ | 0.000 | 0.040 | 0.174 | 0.199 | 0.408^*^ | 0.392 | 0.715^**^ | 0.270 |
|  | SDZ | 0.057 | 0.166 | 0.104 | 0.084 | 0.303 | 0.271 | 0.455^*^ | 0.194 |
|  | TC | 0.028 | -0.139 | 0.236 | 0.034 | -0.033 | -0.082 | 0.557^**^ | -0.151 |
|  | OTC | 0.000 | -0.082 | 0.146 | -0.032 | -0.138 | -0.180 | 0.469^*^ | -0.205 |
|  | CTC | 0.045 | 0.143 | 0.438^*^ | -0.110 | -0.127 | -0.086 | 0.110 | -0.205 |
|  | *intI1* | 0.318 | 0.566^**^ | 0.127 | 0.356 | 0.638^**^ | 0.681^**^ | 0.210 |  |

**Table S5. The relative abundance of antibiotic resistance genes in plant materials.** G refers to groundwater, W refers to livestock wastewater, C refers to conventional furrow irrigation, A refers to alternate-furrow irrigation. 100, 50, 65 and 80 refer to 100%, 50%, 65% and 80% of full irrigation amount per plot, respectively.

|  |  | *tetA* | *tetG* | *tetO* | *tetW* | *tetX* | *sulI* | *sulII* |
| --- | --- | --- | --- | --- | --- | --- | --- | --- |
| GC100 | Roots | 1.000 | 1.000 | 1.000 | 1.000 | 1.000 | 1.000 | 1.000 |
|  | Stems | 1.901 | 0.082 | 0.002 | 0.270 | 0.530 | 0.114 | 0.043 |
|  | Leaves | 1.074 | 0.105 | 0.001 | 0.140 | 0.028 | 0.036 | 0.007 |
|  | Fruits | 0.291 | 0.018 | 0.001 | 0.017 | 0.032 | 0.005 | 0.000 |
| GA50 | Roots | 1.935 | 0.485 | 0.766 | 0.361 | 5.893 | 0.320 | 0.777 |
|  | Stems | 1.017 | 0.084 | 0.132 | 0.284 | 0.098 | 0.045 | 0.018 |
|  | Leaves | 1.212 | 0.095 | 0.003 | 0.184 | 0.125 | 0.048 | 0.008 |
|  | Fruits | 0.366 | 0.025 | 0.000 | 0.022 | 0.016 | 0.002 | 0.000 |
| GA65 | Roots | 0.011 | 1.011 | 0.283 | 0.928 | 3.261 | 0.232 | 0.039 |
|  | Stems | 1.747 | 0.051 | 0.001 | 0.194 | 0.220 | 0.072 | 0.037 |
|  | Leaves | 0.530 | 0.057 | 0.004 | 0.135 | 0.145 | 0.025 | 0.006 |
|  | Fruits | 0.314 | 0.014 | 0.000 | 0.004 | 0.071 | 0.002 | 0.001 |
| GA80 | Roots | 0.290 | 0.992 | 0.193 | 0.608 | 1.786 | 0.188 | 0.038 |
|  | Stems | 0.300 | 0.020 | 0.000 | 0.072 | 0.224 | 0.026 | 0.027 |
|  | Leaves | 2.172 | 0.193 | 0.004 | 0.528 | 0.170 | 0.100 | 0.024 |
|  | Fruits | 0.249 | 0.016 | 0.000 | 0.017 | 0.020 | 0.002 | 0.000 |
| WC100 | Roots | 1.381 | 1.358 | 0.107 | 1.208 | 30.208 | 0.286 | 0.023 |
|  | Stems | 0.351 | 0.035 | 0.000 | 0.072 | 3.220 | 0.024 | 0.008 |
|  | Leaves | 6.151 | 0.068 | 0.003 | 0.226 | 2.722 | 0.074 | 0.034 |
|  | Fruits | 0.203 | 0.017 | 0.001 | 0.018 | 0.640 | 0.019 | 0.001 |
| WA50 | Roots | 1.522 | 4.533 | 0.180 | 0.673 | 48.511 | 0.509 | 0.054 |
|  | Stems | 2.128 | 0.136 | 0.005 | 0.387 | 9.368 | 0.088 | 0.004 |
|  | Leaves | 0.999 | 0.063 | 0.006 | 0.245 | 4.371 | 0.079 | 0.011 |
|  | Fruits | 0.257 | 0.011 | 0.000 | 0.013 | 2.695 | 0.016 | 0.003 |
| WA65 | Roots | 0.270 | 0.778 | 0.018 | 0.059 | 62.845 | 0.226 | 0.009 |
|  | Stems | 0.442 | 0.050 | 0.002 | 0.083 | 5.444 | 0.024 | 0.014 |
|  | Leaves | 0.096 | 0.008 | 0.001 | 0.042 | 0.775 | 0.009 | 0.001 |
|  | Fruits | 0.062 | 0.003 | 0.000 | 0.001 | 0.337 | 0.001 | 0.001 |
| WA80 | Roots | 0.453 | 1.553 | 0.170 | 0.473 | 26.728 | 0.145 | 0.031 |
|  | Stems | 0.728 | 3.813 | 0.077 | 1.596 | 3.896 | 0.893 | 0.434 |
|  | Leaves | 0.379 | 0.049 | 0.003 | 0.088 | 0.270 | 0.066 | 0.002 |
|  | Fruits | 0.118 | 0.001 | 0.000 | 0.003 | 0.175 | 0.002 | 0.001 |

**Table S6. Concentration of antibiotic compounds (ng g^-1^) in pepper plants grown under different irrigation regimes.** G refers to groundwater, W refers to livestock wastewater, C refers to conventional furrow irrigation, A refers to alternate-furrow irrigation. 100, 50, 65 and 80 refer to 100%, 50%, 65% and 80% of full irrigation amount per plot, respectively.

|  | Chlortetracycline | | | |  | | Tetracycline | | | |  | Oxytetracycline | | | |
| --- | --- | --- | --- | --- | --- | --- | --- | --- | --- | --- | --- | --- | --- | --- | --- |
|  | Roots | Stems | Leaves | Fruits |  | Roots | | Stems | Leaves | Fruits |  | Roots | Stems | Leaves | Fruits |
| GC100 | 8.34 | 12.51 | 5.28 | 10.09 |  | 3.33 | | 8.13 | 3.40 | 8.30 |  | 4.84 | 7.21 | 4.36 | 6.79 |
| GA50 | 4.84 | 8.87 | 4.66 | 7.87 |  | 2.29 | | 8.39 | 3.89 | 6.62 |  | 4.07 | 6.49 | 4.59 | 5.62 |
| GA65 | 5.69 | 6.02 | 3.53 | 5.11 |  | 2.40 | | 3.52 | 3.99 | 5.46 |  | 3.76 | 5.44 | 4.31 | 6.44 |
| GA80 | 1.31 | 3.88 | 2.42 | 7.09 |  | 2.30 | | 4.39 | 3.83 | 5.38 |  | 4.05 | 6.93 | 3.46 | 5.39 |
| WC100 | 5.54 | 4.51 | 1.84 | 6.10 |  | 6.11 | | 7.53 | 8.90 | 14.55 |  | 8.58 | 6.93 | 8.63 | 12.67 |
| WA50 | 3.25 | 1.58 | 1.28 | 5.06 |  | 5.73 | | 7.15 | 8.45 | 15.44 |  | 5.90 | 6.17 | 7.86 | 12.97 |
| WA65 | 2.81 | 9.21 | 1.18 | 5.05 |  | 5.68 | | 8.05 | 8.80 | 15.66 |  | 5.33 | 6.50 | 7.77 | 16.90 |
| WA80 | 1.80 | 1.74 | 1.65 | 3.92 |  | 5.70 | | 6.71 | 8.48 | 14.94 |  | 5.65 | 5.84 | 7.71 | 13.09 |
|  | Sulfamethoxazole | | | |  | Sulfamerazine | | | | |  | Sulfadiazine | | | |
|  | Roots | Stems | Leaves | Fruits |  | Roots | | Stems | Leaves | Fruits |  | Roots | Stems | Leaves | Fruits |
| GC100 | 0.54 | 2.22 | 0.18 | 0.90 |  | 2.05 | | 3.61 | 1.26 | 4.31 |  | 0.77 | 1.60 | 0.75 | 1.71 |
| GA50 | 0.85 | 1.04 | 0.26 | 0.14 |  | 1.80 | | 2.70 | 1.57 | 2.20 |  | 0.65 | 1.30 | 0.81 | 0.97 |
| GA65 | 1.14 | 0.67 | 0.15 | 0.20 |  | 1.22 | | 1.89 | 2.40 | 2.67 |  | 0.51 | 0.81 | 1.17 | 1.05 |
| GA80 | 0.39 | 0.53 | 0.08 | 0.32 |  | 1.19 | | 1.60 | 1.73 | 4.32 |  | 0.50 | 0.86 | 0.86 | 1.37 |
| WC100 | 6.03 | 3.21 | 5.92 | 6.79 |  | 4.57 | | 3.80 | 4.17 | 9.80 |  | 1.60 | 1.77 | 2.29 | 3.98 |
| WA50 | 3.32 | 3.43 | 5.21 | 6.59 |  | 4.33 | | 4.72 | 4.82 | 9.86 |  | 1.58 | 1.81 | 2.28 | 4.11 |
| WA65 | 2.86 | 3.25 | 8.46 | 7.58 |  | 3.21 | | 4.28 | 4.83 | 9.81 |  | 1.59 | 1.87 | 2.30 | 4.55 |
| WA80 | 2.86 | 4.61 | 8.67 | 6.40 |  | 2.52 | | 4.21 | 4.29 | 9.05 |  | 1.50 | 1.78 | 2.17 | 3.85 |
